# Enhanced production of polyunsaturated fatty acids by enzyme engineering of tandem acyl carrier proteins

**DOI:** 10.1038/srep35441

**Published:** 2016-10-18

**Authors:** Shohei Hayashi, Yasuharu Satoh, Tetsuro Ujihara, Yusuke Takata, Tohru Dairi

**Affiliations:** 1 Graduate School of Chemical Sciences and Engineering, Hokkaido University, N13-W8, Kita-ku, Sapporo 060-8628, Japan; 2 Graduate School of Engineering, Hokkaido University, N13-W8, Kita-ku, Sapporo 060-8628, Japan; 3 Kyowa Hakko Bio Co. Ltd., 1-6-1, Ohtemachi, Chiyoda-ku, Tokyo 100-8185, Japan; 4 Research Faculty of Agriculture, Hokkaido University, N9-W9, Kita-ku, Sapporo 060-8589, Japan

## Abstract

In some microorganisms, polyunsaturated fatty acids (PUFAs) are biosynthesized by PUFA synthases characterized by tandem acyl carrier proteins (ACPs) in subunit A. These ACPs were previously shown to be important for PUFA productivity. In this study, we examined their function in more detail. PUFA productivities increased depending on the number of ACPs without profile changes in each subunit A of eukaryotic and prokaryotic PUFA synthases. We also constructed derivative enzymes from subunit A with 5 × ACPs. Enzymes possessing one inactive ACP at any position produced ~30% PUFAs compared with the parental enzyme but unexpectedly had ~250% productivity compared with subunit A with 4 × ACPs. Enzymes constructed by replacing the 3^rd^ ACP with an inactive ACP from another subunit A or ACP-unrelated sequences produced ~100% and ~3% PUFAs compared with the parental 3^rd^ ACP-inactive enzyme, respectively. These results suggest that both the structure and number of ACP domains are important for PUFA productivity.

Polyunsaturated fatty acids (PUFAs, Fig. 1a) including docosahexaenoic acid (DHA; C22:6 ω3), eicosapentaenoic acid (EPA; C20:5 ω3), and arachidonic acid (ARA; C20:4 ω6) are essential components of membrane lipids and for human nutrition. They also have crucial biological activities such as prevention of arteriosclerosis and hyperlipidaemia^1^,^2^,^3^,^4^. Therefore, the demand for PUFAs is increasing as medical pharmaceuticals and nutritional supplements.

Fishes and fish oils have traditionally been the sole PUFA sources. However, there is concern over the availability of PUFAs because of the unstable supply from marine resources and increasing demand. Therefore, alternative and sustainable sources of PUFAs are required. To this end, fermentative processes have been developed using microorganisms such as microalgae, fungi, and engineered yeasts for the production of DHA, ARA, and EPA, respectively^5^,^6^,^7^.

PUFAs are biosynthesized by two pathways, the aerobic desaturase/elongase pathway and the anaerobic PUFA synthase pathway. In the former pathway, which operates in plants, fungi, microalgae, and bacteria, specific desaturases and elongases catalyse individual desaturation and elongation steps to synthesize PUFAs from oleic acid (C18:1 ω9)^7^. In the latter pathway, which occurs in eukaryotic microalgae and prokaryotic bacteria, PUFA synthases composed of huge enzyme complexes with multiple catalytic domains synthesize PUFAs using acetyl coenzyme A (CoA) and malonyl-CoA as starter and extender units, respectively, in a manner similar to polyketide synthases (PKSs) and fatty acid synthases (FASs)^8^,^9^,^10^,^11^. From the viewpoint of industrial production of PUFAs, the latter process has great advantages because it requires fewer reducing equivalents such as NADPH and produces smaller amounts of by-products with undesirable chain lengths and unsaturated positions.

PUFA synthase genes have been identified as clusters not only in marine microorganisms such as *Shewanella oneidensis*^12^, *Photobacterium profundum*^13^, *Moritella marina*^14^, *Aureispira marina*^15^, and *Schizochytrium* sp.^16^, but also in terrestrial myxobacteria^17^. All the PUFA synthases identified are huge multifunctional enzyme complexes consisting of three to four subunits. They possess acyltransferase (AT), malonyl-CoA transferase (MAT), ketoacyl synthase (KS), ketoacyl reductase (KR), dehydratase (DH), enoyl reductase (ER), chain length factor (CLF), and acyl carrier protein (ACP) domains, and all domains are involved in the elongation cycle for PUFA biosynthesis (Fig. 1b,c). PUFA synthases have unique features distinct from other PKSs. Their DH domains show similarity to that of FabA, which catalyses *cis* double bond formation via dehydration and isomerization in fatty acid biosynthesis. Therefore, these domains perhaps participate in the introduction of *cis* configurations in PUFA biosynthesis^9^. In addition, PUFA synthases have from four to nine multi-tandem ACP domains. After heterologous expression of PUFA genes in *Escherichia coli* was successfully achieved^12^,^15^,^16^,^18^, Jiang *et al*. examined the biological role of these ACP domains using this system and PKSs with site-directed mutagenized ACP domains. They showed that PUFA productivity decreased depending on the number of inactivated ACP domains^19^.

Modular and iterative type I PKSs with tandem-repeated ACP domains have also been identified and their biological roles have been studied. First, one of the double ACPs in a PKS responsible for naphthopyrone biosynthesis was shown to be enough to produce naphthopyrone, although the effect of inactivated ACPs on productivity was not reported^20^. Thereafter, the double and triple ACPs in PKS and PKS-nonribosomal peptide synthase for mupirocin^21^ and curacin^22^ biosynthesis, respectively, were inactivated by site-directed mutagenesis and in-frame deletion. In both the cases, the mutated enzymes produced lesser amounts of the products than the native types.

These results suggest that the number of multi-tandem ACP domains in PUFA synthases/PKSs and polyketide productivity have a close relationship. However, only disruption methods have been employed in all preceding studies and there have been no reports using a theoretically reverse approach; that is, increasing the number of active ACP domains. In this paper, we constructed PUFA synthase derivatives with more active ACP domains than the native type and examined the effects on productivity. Finally, we succeeded in drastically enhancing PUFA productivity more than 16-fold for EPA and 1.8-fold for DHA. To the best of our knowledge, this is the first example of enhancement of PUFA productivity by enzymatic engineering with the huge enzyme complex PUFA synthase.

## Results

### Biological function of the tandem ACP domains in OrfA

**Figure 2:**
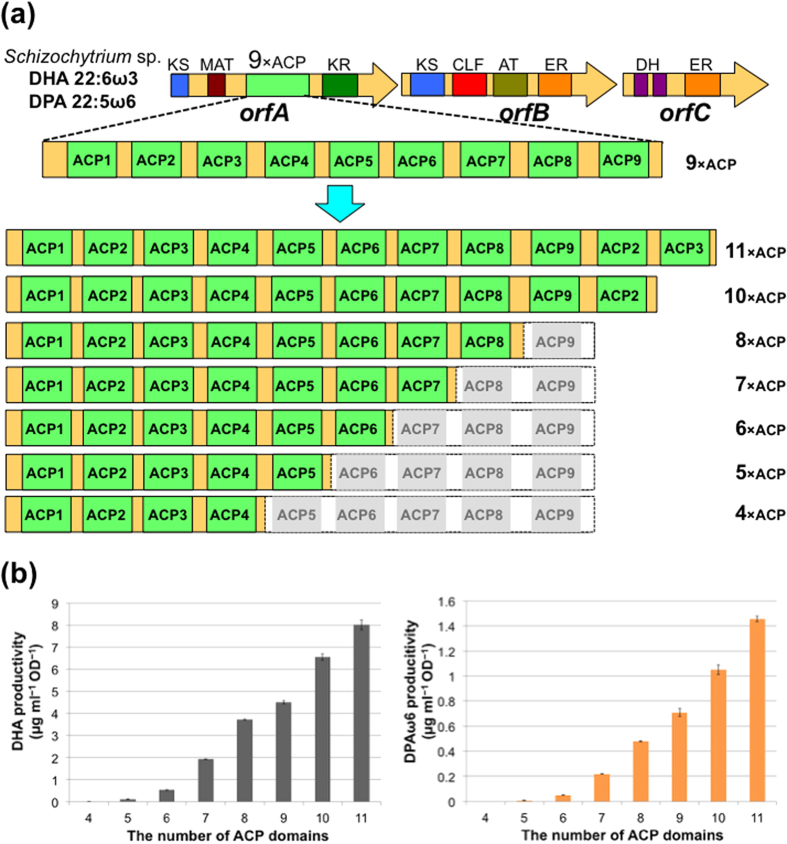
DHA and DPAω6 production by the engineered *orfA*s with *orfBC* and *hetI* in *Escherichia coli.* (**a**) Schematic illustration of engineered *orfA*s with 4× to 11 × ACP domains. (**b**) DHA and DPAω6 productivities by the engineered *orfA*s. Data are presented as mean values with error bars indicating standard deviation from three independent experiments.

We first investigated the effect of increasing and decreasing the number of ACP domains in OrfA of *Schizochytrium* sp. on PUFA synthase activity. PUFA synthase activity was evaluated on the basis of PUFA productivity of recombinant *E. coli* expressing PUFA biosynthetic genes because PUFA synthases are huge enzyme complexes and hence it is difficult to prepare them as recombinants for *in vitro* assay. Metz *et al*. succeeded in producing PUFAs in recombinant *E. coli* harbouring the *orfABC* genes from *Schizochytrium* sp. and phosphopantetheinyl transferase gene, *hetI*, from *Nostoc* sp.^16^ We therefore employed the same strategy. The *orfABC* and *hetI* genes were independently cloned into different and compatible expression vectors to construct pET-*orfA*, pCDF-*orfB*, pCOLA-*orfC*, and pSTV-*hetI*, respectively (Supplementary Methods and Supplementary Fig. 1). To prevent degradation of the synthesized PUFAs, the *fadE* gene encoding an acyl-CoA dehydrogenase, a gene responsible for the β-oxidation pathway in *E. coli* BLR(DE3), was disrupted (Supplementary Methods and Supplementary Fig. 2). After *E. coli* BLR(DE3)*∆fadE* harbouring all the plasmids was cultured in terrific broth medium, the products were analysed by GC/MS. As shown in Supplementary Fig. 3, DHA and DPAω6 were successfully detected. The yields of DHA and DPAω6 were 4.5 ± 0.08 and 0.71 ± 0.03 μg mL^−1^ OD^−1^, respectively, showing that the system constructed in this study worked well under the experimental conditions we employed (Fig. 2b and Supplementary Table 1).

We then constructed pET-*orfA* derivative plasmids with different numbers of ACP domains. Each of the ACP domains is highly conserved and separated by conserved and repeated regions with Ala and Pro rich sequences (Supplementary Fig. 4). This architecture suggested that the regions between the Ala/Pro rich sequences are each one functional ACP unit in the multi-tandem ACP domain of PUFA synthase. Therefore, we increased the number of units in a stepwise manner by the method shown in the Supplementary Methods and Supplementary Fig. 5. In brief, we first constructed a plasmid with one unit of the ACP domain. Then, this unit was inserted into each plasmid by step-by-step addition to construct all the plasmids. Consequently, we constructed seven plasmids with 4×, 5×, 6×, 7×, 8×, 10× and 11 × ACP domains (Fig. 2a). Using these plasmids, the PUFA productivity and profile were analysed as described above. As shown in Fig. 2b and Supplementary Table 1, DHA and DPAω6 productivity decreased and increased depending on the number of ACP domains, although the profiles of the PUFA products were the same as those of the native type (9 × ACP domains) in Supplementary Fig. 3. Notably, the PUFA yields of pET-*orfA* with more ACP domains (10× and 11×) were higher than those of the native plasmid, pET-*orfA* (1.5-fold DHA and DPAω6 for 10 × ACPs, 2.0-fold DPAω6 and 1.8-fold DHA for 11 × ACP). These results indicated that the number of ACP domains in OrfA just controls productivity, and that the tandem ACP domain is a key factor controlling PUFA productivity.

### Effect of the number of ACP domains in SoPfaA on PUFA productivity

**Figure 3:**
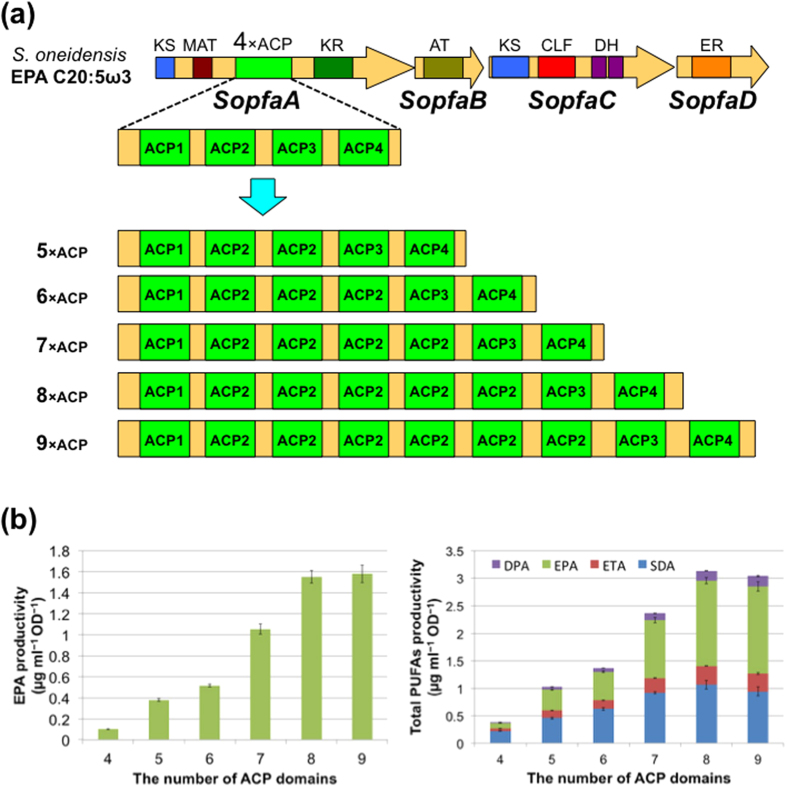
Polyunsaturated fatty acid production by the engineered *SopfaA*s with *SopfaBCDE.* (**a**) Schematic illustration of the increased number of ACP domains. (**b**) EPA and total PUFA productivities by the engineered *SopfaA* with 4× to 9 × ACP domains. Data are presented as mean values with error bars indicating standard deviation from four independent experiments.

To examine whether the functions of ACP domains demonstrated above are a common feature in other PUFA synthases, we next carried out the same experiment with the EPA synthase genes, *SopfaABCDE*, of *Shewanella oneidensis*^12^. The *SopfaA* (with 4 × ACP domains), *C*, and *D* genes were independently cloned into three expression vectors as shown in the Supplementary Methods and Supplementary Fig. 6. The *SopfaB* and *E* genes were inserted into different multi-cloning sites in a pACYCDuet derivative to express them independently. The plasmids were introduced into *E. coli* BLR(DE3)*∆fadE* and the products were analysed by GC/MS. EPA and DPAω3 were successfully detected (Supplementary Fig. 7) with yields of 0.10 ± 0.002 and 0.013 ± 0.003 μg mL^−1^ OD^−1^, respectively (Fig. 3b and Supplementary Table 2). However, stearidonic acid (SDA, C18:4 ω3) was the major product (0.22 ± 0.02 μg mL^−1^ OD^−1^) and eicosatetraenoic acid (ETA, C20:4 ω3, 0.044 ± 0.004 μg mL^−1^ OD^−1^) was also produced as a minor product. Because these PUFAs are not produced by the original strain, *S. oneidensis*, their production was caused by the heterologous expression in *E. coli*. Thus, the *SopfaABCDE* genes were also shown to work under the experimental conditions employed.

We then constructed five plasmids carrying *SopfaA* genes (Fig. 3a) with increased numbers of ACP domains (5×, 6×, 7×, 8×, and 9×) by essentially the same method used to construct the pET-*orfA* derivatives (Supplementary Methods and Supplementary Fig. 8). The productivities and profiles of the PUFAs produced by *E. coli* BLR(DE3)*∆fadE* carrying the *SopfaA* derivative genes together with the *SopfaBCDE* were analysed as described above. As shown in Fig. 3b and Supplementary Table 2, EPA productivity significantly increased with increased numbers of ACP domains (5 × ACPs, 3.7-fold; 6 × ACPs, 5.1-fold; 7 × ACPs, 10-fold; 8 × ACPs, 16-fold; 9 × ACPs, 16-fold). SDA, ETA, and DPAω3 productivities also increased in the same manner. As for the profiles of the PUFA products, no differences between the *SopfaA* derivatives and the native gene were observed (Supplementary Fig. 7). These results again indicated that the number of ACP domains just controls the productivity.

### Effect of inactivation of ACP domain in PfaA on PUFA productivity

As demonstrated by the PUFA synthases of both *Schizochytrium* sp. and *S. oneidensis*, which are a eukaryotic alga and a prokaryotic microorganism, respectively, an increased number of ACP domains up to 9 or 11 linearly enhanced PUFA productivity, suggesting that more ACP domains plausibly supply more substrates to synthesize PUFAs. Therefore, we next investigated the effect of inserting an inactive ACP domain, in which the active Ser residue was changed to Ala by site-directed mutagenesis, on PUFA productivity.

**Figure 4:**
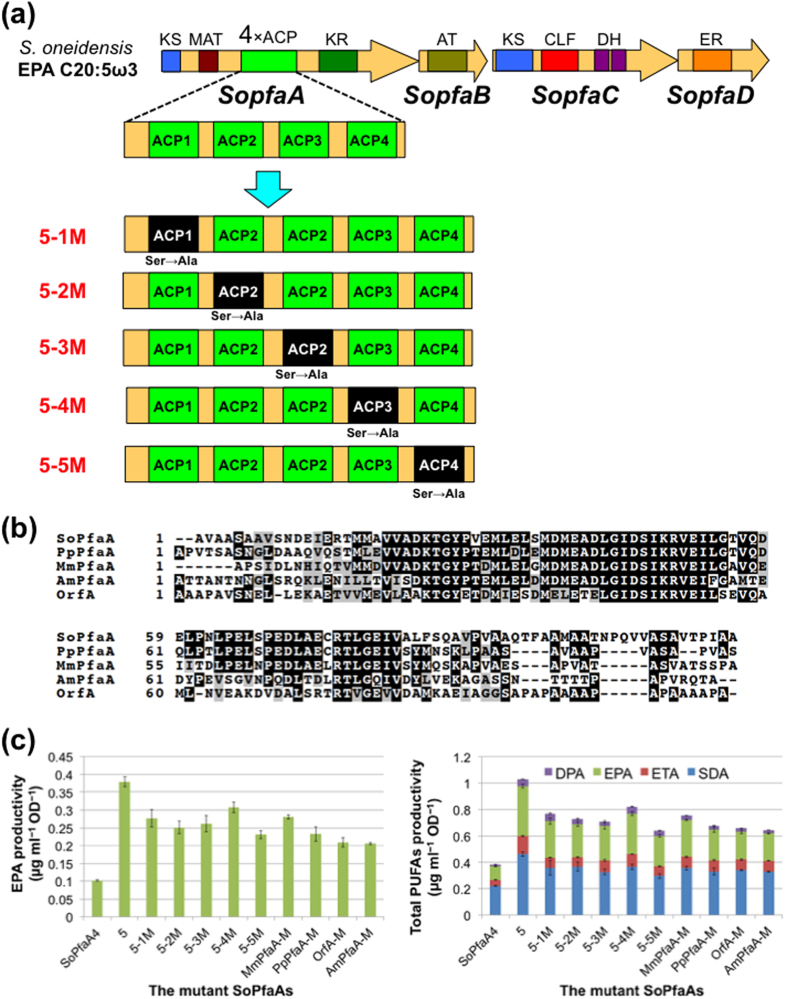
Polyunsaturated fatty acid production by mutant *SopfaA*s with *SopfaBCDE*. (**a**) Schematic illustration of mutated *SopfaA*s. (**b**) Sequence alignment of the ACP domain of OrfA with those of other PUFA synthases. (**c**) EPA and total PUFA productivities by the mutated *SopfaA*s. Data are presented as mean values with error bars indicating standard deviation from four independent experiments.

We constructed *SopfaA5-3M*, which had the same gene structure as *SopfaA* with 5 × ACP domains except that the third ACP domain was inactivated by replacing the active Ser residue with Ala (Fig. 4a and Supplementary Methods). The PUFA productivity and profile was investigated as described above. As shown in Fig. 4c and Table 1, the transformants harbouring *SopfaA5-3M* produced approximately 30% EPA and total PUFAs compared with those harbouring *SopfaA5*, but unexpectedly produced 260% EPA and 190% total PUFAs compare with those harbouring native SoPfaA with 4 × ACP domains. This result suggested that PUFA productivity was enhanced not only by increasing the number of active ACP domains but also by the insertion of an inactivated ACP domain. To better understand this unexpected result, we constructed an additional four plasmids, *SopfaA5-1M*, *SopfaA5-2M*, *SopfaA5-4M*, and *SopfaA5-5M*, in which the first, second, fourth and fifth active Ser residues of the ACP domain were mutated to Ala, respectively (Fig. 4a and Supplementary Methods). The productivities of the mutated plasmids decreased to 18–39% EPA and 20–38% total PUFAs compared with *SopfaA5*, but increased to 230–300% EPA and 170–220% total PUFAs compared with the native *SopfaA4* (4 × ACP) (Fig. 4c and Table 1) This result suggested that the insertion of inactivated ACP domains could also enhance the yield, although the effects were smaller than those of active ACP domains, and that the location of the inactivated ACP in the tandem ACP domain region is not critical.

To deepen our understanding of this phenomenon, we constructed additional *SopfaA5* derivatives. The inactivated ACP domain located at the third position of *SopfaA5-3M* was replaced with another inactive ACP domain of PUFA synthase from *Moritella marina* (a DHA producer), *Photobacterium profundum* (an EPA producer), *Schizochytrium* sp., or *Aureispira marina* (an ARA producer). Their ACP domains show 70%, 71%, 49% and 48% identities to that of SoPfaA (Fig. 4b and Supplementary Table 3). The procedures for plasmid construction and inactivation of the active site were the same as those described above (Supplementary Methods). Each of the constructed plasmids, *SopfaA-MmpfaA-M*, *SopfaA-PppfaA-M*, *SopfaA-orfA-M*, and *SopfaA-AmpfaA-M*, was introduced into *E. coli* BLR(DE3)*∆fadE* together with the *SopfaBCDE* genes and PUFA productivity was examined. All the enzymes produced similar amounts of PUFAs but the productivities varied slightly depending on the similarity between the native and replaced ACP domains (Fig. 4c and Table 1; 2.8- (EPA) and 2.0-fold (total PUFAs) for MmPfaA-M, 2.3- and 1.8-fold for PpPfaA-M, 2.1- and 1.7-fold for OrfA-M, and 2.0- and 1.7-fold for AmPfaA-M). This result suggested that the structure of the tandem ACP domains is also a key factor controlling PUFA productivity in addition to the number of active ACP domains.

Next, we constructed two additional *SopfaA5* derivatives in a manner similar to those described above (Supplementary Methods). In this case, however, DNA (amino acid) sequences that had no relation to PUFA synthase were used. The ACP domain located at the third position of *SopfaA5-3M* was replaced in-frame with DNA that encoded S1 or S2 of the ABC transporter HlyB protein identified in the *S. oneidensis* genome database and had approximately the same length as the native ACP (Supplementary Table 4). In both cases, PUFA productivity was mostly lost (Table 1).

### Production of high-titre ARA using modified PfaA

**Figure 5:**
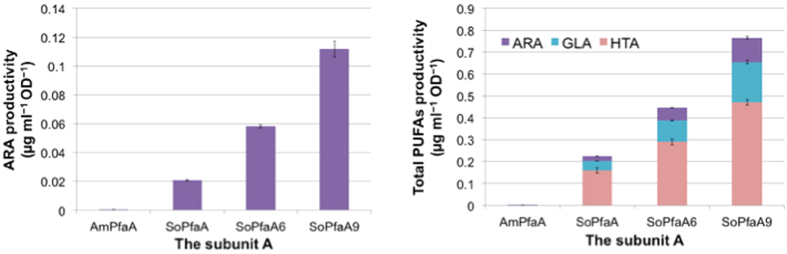
ARA and total polyunsaturated fatty acid productivities by *AmpfaA* and *SopfaA*, 6, and 9 with *AmpfaBCD*. Data are presented as mean values with error bars indicating standard deviation from four independent experiments.

PUFA synthase is composed of three or four subunits, among which the A subunits such as OrfA and PfaA have the same domain structure. Previously, Jiang *et al*. showed that PfaA was not responsible for determination of the PUFA product profile^19^. Additionally, we showed that the ACP domains in PfaA just controlled PUFA productivity in this study. These results suggested that high production of PUFAs could be achieved by replacing a subunit A possessing fewer ACP domains with another one possessing more ACP domains. We examined the plausibility of this using the ARA biosynthetic gene from *A. marina*. Recently, we succeeded in heterologously expressing the five ARA biosynthetic genes, *AmpfaA* (with 4 × ACP domains), *B*, *C*, *D,* and *E*, in *E. coli* although the productivity was quite low^15^. Therefore, we tried enhancing the ARA productivity using another subunit A such as *SopfaA* for EPA biosynthesis. Because the substitution of *AmpfaA* with *SopfaA* in the previously constructed plasmid was technically difficult, we constructed plasmids with the same expression vectors and strategies used for EPA production in this study (Supplementary Methods). However, the productivity of ARA was again quite low even with the new constructs (Fig. 5, Supplementary Fig. 9 and Supplementary Table 5).

Next, we evaluated ARA productivity with *SopfaA*, *SopfaA6* and *SopfaA9* instead of the native *AmpfaA*. As shown in Fig. 5, Supplementary Fig. 9 and Supplementary Table 5, ARA productivity was drastically increased by the simple substitution of *AmpfaA* with *SopfaA* (48-fold), which has the same number of ACP domains as *AmpfaA* (4×). Moreover, *SopfaA6* and *SopfaA9*, which had 6× and 9 × ACP domains, respectively, enhanced the productivity depending on the number of ACP domains (2.8-fold for 6× and 5.5-fold for 9× compared with *SopfaA*), and also enhanced the productivities of γ-linoleic acid (GLA, C18:3 ω6) and 4,7,10-hexadecatrienoic acid (HTA, C16:3 ω6; Supplementary Fig. 10). These results showed that the substitution of subunit A with another native one with high activity and/or with engineered ones possessing an increased number of ACP domain is a useful strategy for high PUFA production.

## Discussion

To supply PUFAs stably, fermentation approaches using microorganisms have been developed. Among PUFAs, ARA and DHA are industrially produced by the engineered aerobic desaturase/elongase pathway of fungi and the anaerobic PUFA synthase pathway of algae, respectively. However, genetic engineering of these microorganisms is usually difficult and alternative heterologous expression systems have been used to survey the bottleneck of PUFA biosynthesis. In the case of the aerobic desaturase/elongase pathway, improvement of reducing equivalent (NADPH) flux, utilization of inhibiters of the acyl-exchange reaction between phosphatidylcholine and acyl-CoA substrates, and prevention of PUFA degradation by β-oxidation were shown to be effective for titre improvement^6^,^23^. For anaerobic PUFA synthase, co-expression of catalase^24^, addition of cerulenin^25^,^26^, an inhibiter of *de novo* fatty acid synthesis, and metabolic engineering to increase the substrate supply^27^ have been employed. However, all these attempts have focussed only on the metabolic flow and no examples of activation of enzyme activity have been reported. In this study, we succeeded in improving PUFA productivity for the first time by enzymatic engineering.

PUFA productivities were almost linearly increased depending on the number of ACP domains with both the eukaryotic PUFA synthase of *Schizochytrium* sp. and prokaryotic PUFA synthase of *S. oneidensis*. However, the PUFA product profiles of the engineered enzymes were the same as those of the native type. Taking the reaction mechanisms of these PUFA synthases together, we speculated that tandem ACP domains would allow for simultaneous access of other enzyme domains and enhance productivity. To test this hypothesis, we constructed five mutants (SoPfaA5-1M to SoPfaA5-5M) in which the first to fifth active Ser residues of the ACP domain were mutated to Ala, respectively. All the mutants produced 20–37% PUFAs compared with the parental enzyme (SoPfaA5). However, the amounts were unexpectedly higher than that of SoPfaA4 possessing four active ACP domains (Fig. 4 and Table 1), suggesting that the structure of the tandem ACP domains is also a key factor controlling PUFA productivity besides the number of active ACP domains.

The solution structure of the five tandem ACP domains of PUFA synthase was previously investigated using several analytical methods^28^. Small-angle X-ray scattering analysis suggested the multi-ACP fragment was an elongated monomer with a beads-on-a-string like structure. Our abovementioned results with mutated enzymes might support this model. However, gel filtration of the five-tandem-ACP domain showed a shorter retention time than expected from the molecular weight. This result suggested the formation of an oligomeric quaternary structure for the tandem ACP domain although it was concluded that the phenomenon was caused by an elongated protein shape. Our additional mutated enzymes (SoPfaA-MmPfaA-M, SoPfaA-PpPfaA-M, SoPfaA-OrfA-M, and SoPfaA-AmPfaA-M) in which the third ACP of SoPfaA5-3M was replaced with an inactive ACP domain from other PUFA synthases support the beads-on-a-string structure because the enzymes produced almost the same amounts of PUFAs. However, the productivities of these enzymes varied slightly depending on the similarity between native and replaced ACP domain. The result might suggest the tandem ACP domains might interact with each other and an oligomeric quaternary structure might indeed be formed as previously suggested by gel filtration^28^.

In conclusion, to investigate the biological function of the tandem ACP domains in PUFA synthases, we constructed PUFA synthase derivatives with less and more active ACP domains than the native enzyme and examined the effects on PUFA productivity. We clearly demonstrated that subunit A participates in PUFA productivity and is exchangeable with other A subunits; in particular, the number of active ACP domains unequivocally controls PUFA productivity. Taking advantage of this, we were able to produce greater amounts of ARA with SoPfaA9 isolated from an EPA producer. We also showed that the structure of subunit A and the amino acid sequences of the ACP domains are important for PUFA productivity. Thus, the engineering of subunit A is a powerful tool to enhance PUFA productivity. This is the first example of molecular breeding to increase PUFA production by enzymatic engineering with the huge enzyme complex PUFA synthase. As a next step, it will be necessary to examine whether our approach is effective even in the original strains. Furthermore, the product profile including the omega position was suggested to be strictly controlled by subunits B, C, and/or D of PUFA synthase but the mechanism remains to be uncovered.

## Methods

### General

Methyl esters of DHA (C22:6 ω3), DPAω3 (C22:5 ω3), DPAω6 (C22:5 ω6), EPA (C20:5 ω3), ETA (C20:4 ω3), vaccenic acid (C18:1 ω7), palmitic acid (C16:0), GLA (C18:3 ω6), SDA (C18:4 ω3), and other chemicals were purchased from Sigma-Aldrich Japan K.K. (Tokyo, Japan) or Cayman Chemical Company (Ann Arbor, MI, USA). Primers were purchased from FASMAC Co. Ltd. (Kanagawa, Japan). Heptadecanoic acid (C17:0) was obtained from Tokyo Chemical Industry Co. Ltd. (Tokyo, Japan). Enzymes and kits for DNA manipulation were purchased from Takara Bio Inc. (Shiga, Japan) or New England Biolabs Japan Inc. (Tokyo, Japan). PCR reactions were carried out using a GeneAmp PCR System 9700 thermal cycler (Thermo Fisher Scientific Inc., Waltham, MA, USA) with Tks Gflex DNA polymerase (Takara Bio). General genetic manipulations of *E. coli* were performed according to standard protocols.

### Bacterial strains and media

The strains used in this study are summarized in Supplementary Table 6. *E. coli* XL1-Blue (Nippon Gene Co. Ltd., Tokyo, Japan) was routinely used for plasmid construction. For PUFA production, a β-oxidation deficient mutant, *E. coli* BLR(DE3)*∆fadE*, was constructed with *E. coli* BLR(DE3) (Merck KGaA, Darmstadt, Germany) and an *E. coli* gene deletion kit (Gene Bridges GmbH, Heidelberg, Germany).

The media used were LB broth medium (Lennox; Sigma-Aldrich Japan) and terrific broth (TB) medium (Becton, Dickinson and Company, Franklin Lakes, NJ, USA). For growth on plates, 1.5% agar was added into the media. Ampicillin (Ap), chloramphenicol (Cm), kanamycin (Km), and streptomycin (Sm) were added to the media at concentrations of 100, 30, 25, and 20 mg L^−1^, respectively, if necessary.

### Plasmid construction

The plasmids used in this study are summarized in Supplementary Table 7. DNA fragments carrying PUFA synthase genes were amplified by PCR with the primers shown in Supplementary Table 8 and genomic DNA of *Schizochytrium* sp. (ATCC 20888, *orfABC*; accession numbers AF378327, AF378328, AF378329), *Shewanella oneidensis* MR-1 (ATCC BAA-1096, *SopfaABCDE*; accession number NC_004347), and *Aureispira marina* (JCM 23201, *AmpfaABCDE*; accession number AB980240). The amplified fragments were digested with appropriate restriction enzymes and inserted into the corresponding restriction sites of the expression vectors. Detailed processes for plasmid construction are described in the Supplementary Methods. To prepare holo-enzymes of OrfA, a plasmid carrying the 4′-phosphopantetheinyl transferase (*hetI*; accession number L22883) gene of *Nostoc* sp. PCC 7120 (ATCC 27893) was constructed^16^. To construct plasmids with modified ACP domains, the ACP genes *MmpfaA* of *Moritella marina* MP-1 (ATCC 15381, accession number AB025342) and *PppfaA* of *Photobacterium profundum* SS9 (ATCC BAA-1252, accession number CR354531) were used.

### PUFA production

To prevent degradation of the synthesized PUFAs, *E. coli* BLR(DE3)∆*fadE* was used as a host. The PUFA biosynthetic gene sets were co-introduced into the host with the corresponding phosphopantetheinyl transferase gene (*hetI* for *orfA*, *SopfaE* for *SopfaA*, or *AmpfaE* for *AmpfaA*). The transformants were cultured at 30 °C in TB broth medium for 24 h, and then 1 mL of the broth was inoculated into 200-mL baffled flasks containing 20 mL of TB medium and 1 mM IPTG. After cultivation for 48 h at 20 °C with agitation (230 rpm), 5 mL of the culture broth were collected and centrifuged. Total lipids were extracted from the pelleted cells following Bligh and Dyer^29^. For methyl esterification, the lipid fraction was dissolved in hexane (1 mL), to which methanol containing 14 wt% boron trifluoride (1 mL, Sigma-Aldrich Japan) was added, and incubated at 60 °C for 10 min. After the reaction mixture was evaporated, the pellet was dissolved with 0.2 mL of hexane and analysed with a Shimadzu GCMS-QP2010 Ultra system (Kyoto, Japan) equipped with a VF-23 ms column (0.25 mm × 60 m, film thickness 0.25 μm, Agilent Technologies Inc., Santa Clara, CA, USA). The analytical conditions were as follows; carrier gas, helium with constant flow rate at 1.4 mL min^−1^; injection temperature, 250 °C; column temperature, 150 °C (5 min) −250 °C (2 °C min^−1^) −250 °C (15 min); ion source temperature, 250 °C; detection, scan mode (*m/z* 50 to 500) for qualitative analysis and selected ion mode (*m/z* 79) for quantitative analysis. PUFA products were identified by comparing mass spectra of the products with those of authentic ones and by utilizing National Institute of Standards and Technology (NIST) mass spectral library. Heptadecanoic acid was used as an internal standard. To determine the double bond positions of the PUFAs, pyrrolidide derivatives of fatty acid methyl esters were prepared^30^ and analysed by GC/MS.

## Additional Information

**How to cite this article**: Hayashi, S. *et al*. Enhanced production of polyunsaturated fatty acids by enzyme engineering of tandem acyl carrier proteins. *Sci. Rep.* **6**, 35441; doi: 10.1038/srep35441 (2016).

## Supplementary Material

Supplementary Information

Supplementary Table7

Supplementary Table8

## Figures and Tables

**Figure 1:**
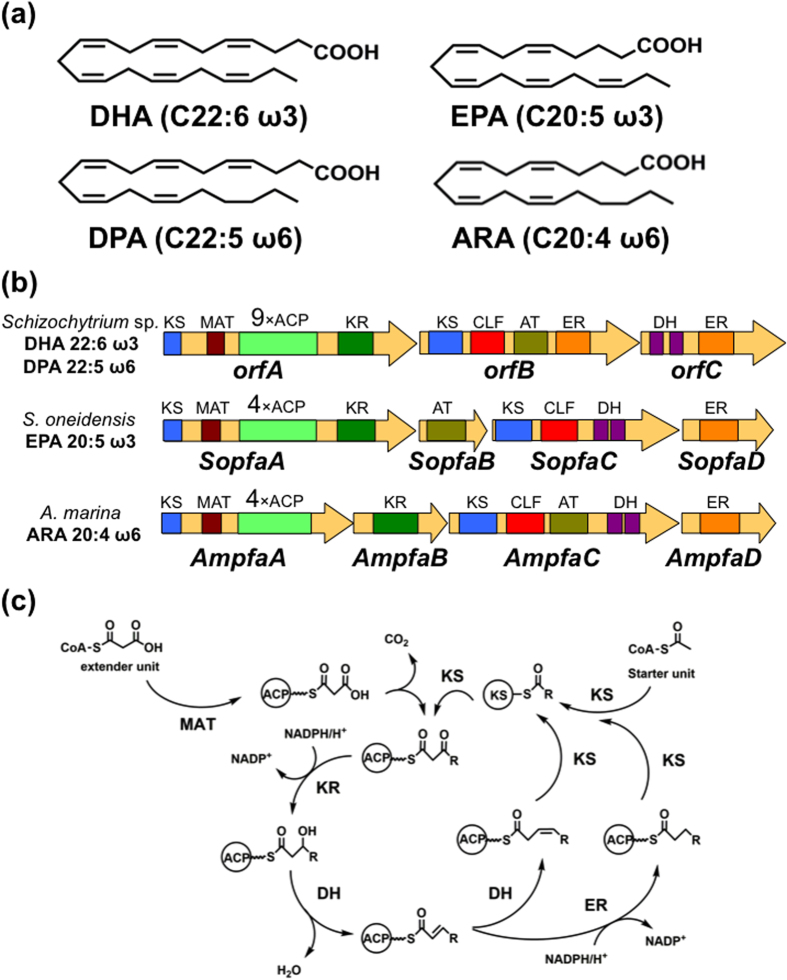
Polyunsaturated fatty acid synthase pathway in microorganisms. (**a**) Chemical structures of DHA, DPAω6, EPA, and ARA. (**b**) Architectures of PUFA synthase gene clusters, KS: ketoacyl synthase, MAT: malonyl CoA transferase, ACP: acyl carrier protein, KR: ketoacyl reductase, CLF: chain length factor, AT: acyltransferase, DH: dehydratase, ER: enoyl reductase. (**c**) Reactions catalysed by PUFA synthases: MAT transfers an extender unit, malonyl-CoA, to ACP; KS attaches the extender unit to the starter unit or acyl-ACP by decarboxylative Claisen condensation; KR reduces the carbonyl group NADPH-dependently; DH and ER catalyse dehydration and NADPH-dependent reduction to form a saturated C-C bond; The FabA-like DH domain perhaps catalyses isomerization.

**Table 1:** Polyunsaturated fatty acid productivity of *Escherichia coli* expressing mutant *SopfaA*s with *SopfaBCD*.

mutant *SopfaAs*	OD_600_	SDA (ratio)* μg ml^−1^ OD^−1^	ETA (ratio)* μg ml^−1^ OD^−1^	EPA (ratio)* μg ml^−1^ OD^−1^	DPAω3 (ratio)* μg ml^−1^ OD^−1^	Total PUFAs**(ratio)* μg ml^−1^ OD^−1^
4 (native)	21.6 ± 1.5	0.22 ± 0.02	0.045 ± 0.003	0.10 ± 0.002	0.013 ± 0.003	0.38 ± 0.01
5	22.2 ± 0.9	0.46 ± 0.02 (2.1)	0.14 ± 0.004 (3.0)	0.38 ± 0.01(3.7)	0.051 ± 0.002 (4.0)	1.0 ± 0.03 (2.7)
5-1M	18.9 ± 0.8	0.36 ± 0.06 (1.6)	0.077 ± 0.004 (1.7)	0.28 ± 0.02 (2.7)	0.056 ± 0.006 (4.3)	0.77 ± 0.08 (2.0)
5-2M	18.6 ± 2.1	0.37 ± 0.03 (1.7)	0.071 ± 0.003 (1.6)	0.25 ± 0.02 (2.5)	0.039 ± 0.003 (3.0)	0.73 ± 0.07 (1.9)
5-3M	20.3 ± 2.1	0.33 ± 0.02 (1.5)	0.087 ± 0.009 (1.9)	0.26 ± 0.02 (2.6)	0.033 ± 0.005 (2.6)	0.71 ± 0.05 (1.9)
5-4M	22.7 ± 1.8	0.37 ± 0.02 (1.7)	0.095 ± 0.002 (2.1)	0.31 ± 0.01 (3.0)	0.053 ± 0.002 (4.1)	0.82 ± 0.03 (2.2)
5-5M	21.6 ± 1.1	0.30 ± 0.02 (1.3)	0.074 ± 0.004 (1.6)	0.23 ± 0.01 (2.3)	0.040 ± 0.004 (3.1)	0.64 ± 0.03 (1.7)
mutant average		0.35 ± 0.03 (1.6)	0.081 ± 0.009 (1.8)	0.27 ± 0.03 (2.6)	0.044 ± 0.009 (3.4)	0.73 ± 0.06 (1.9)
MmPfaA-M	22.1 ± 1.2	0.36 ± 0.01 (1.6)	0.085 ± 0.007 (1.9)	0.28 ± 0.01 (2.8)	0.034 ± 0.001 (2.6)	0.75 ± 0.02 (2.0)
PpPfaA-M	20.8 ± 0.6	0.33 ± 0.03 (1.5)	0.083 ± 0.006 (1.8)	0.23 ± 0.02 (2.3)	0.030 ± 0.003 (2.4)	0.68 ± 0.06 (1.8)
OrfA-M	21.7 ± 1.0	0.34 ± 0.01 (1.5)	0.080 ± 0.007 (1.8)	0.21 ± 0.01 (2.1)	0.028 ± 0.002 (2.2)	0.66 ± 0.02 (1.7)
AmPfaAA-M	20.5 ± 0.5	0.33 ± 0.01 (1.5)	0.082 ± 0.002 (1.8)	0.21 ± 0.003 (2.0)	0.025 ± 0.001 (2.0)	0.64 ± 0.01 (1.7)
S1	20.8 ± 0.8	0.03 ± 0.003	N.D	0.007 ± 0.001	N.D	0.039 ± 0.004
S2	19.3 ± 0.4	0.01 ± 0.001	N.D	N.D	N.D	0.010 ± 0.001

Data are presented as mean values ± standard deviation derived from four independent experiments.

^*^Relative values when the value of native enzyme is taken as 1.

^**^Sum of SDA, ETA, EPA, and DPAω3.
