# Supplementary material for: Enhanced production of polyunsaturated fatty acids by enzyme engineering of tandem acyl carrier proteins
Source: Sci Rep. 2016 Oct 18;6:35441. doi: 10.1038/srep35441 (PMC5067506; doi:10.1038/srep35441)
Supplement: Supplementary Information [file srep35441-s1.docx]

**Supplementary Information**

**Enhanced production of polyunsaturated fatty acids by enzyme engineering of tandem acyl carrier proteins**

Shohei Hayashi, Yasuharu Satoh, Tetsuro Ujihara, Yusuke Takata & Tohru Dairi

*
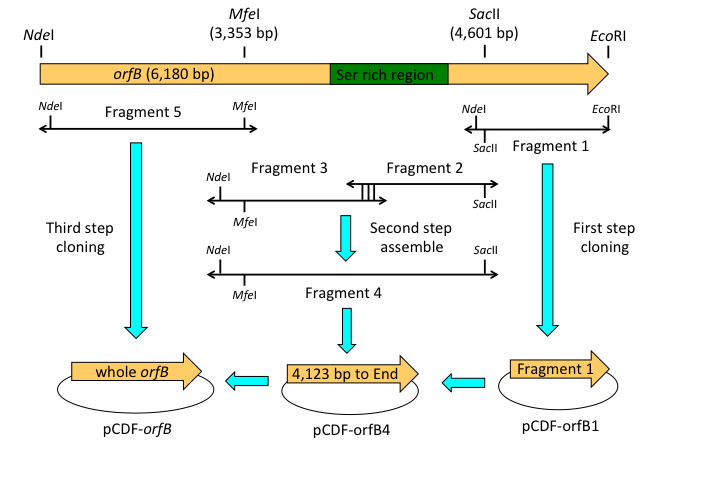
*

**Supplementary Fig. 1. Strategy for pCDF-*orfB* construction.**


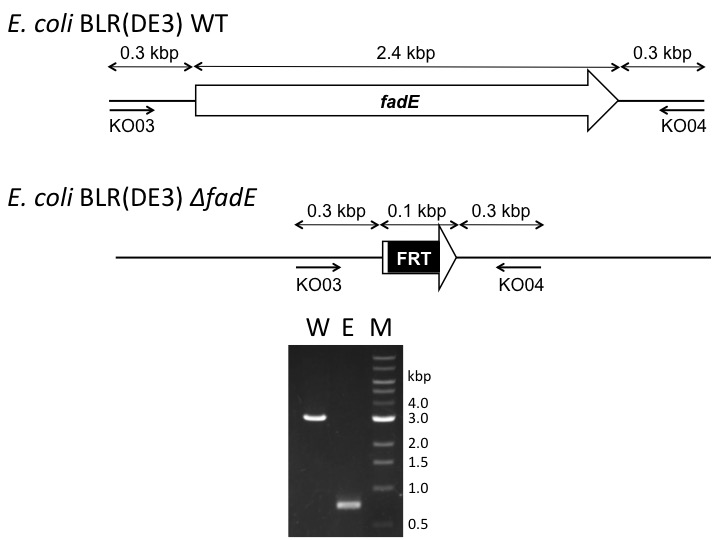


**Supplementary Fig. 2. Deletion of the *fadE* gene in *Escherichia coli* BLR(DE3).** The *fadE* regions of the wild type (upper) and *∆fadE* strain (middle) are shown schematically. Arrows indicate the primers used for PCR analysis. Disruption was confirmed by PCR (bottom), W, wild type; E, *∆fadE* disruptant; M, marker

**(a)**

**
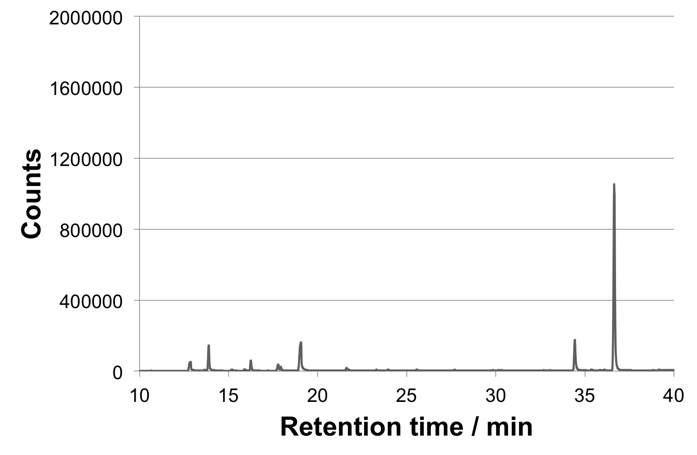

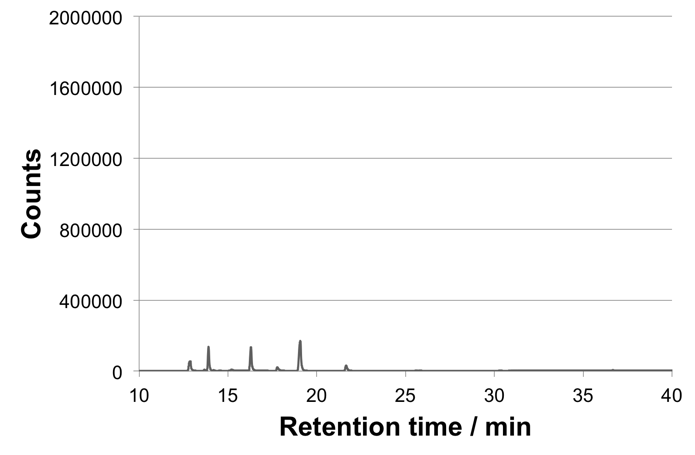
**

**OrfA 4×**

**OrfA 8×**

4
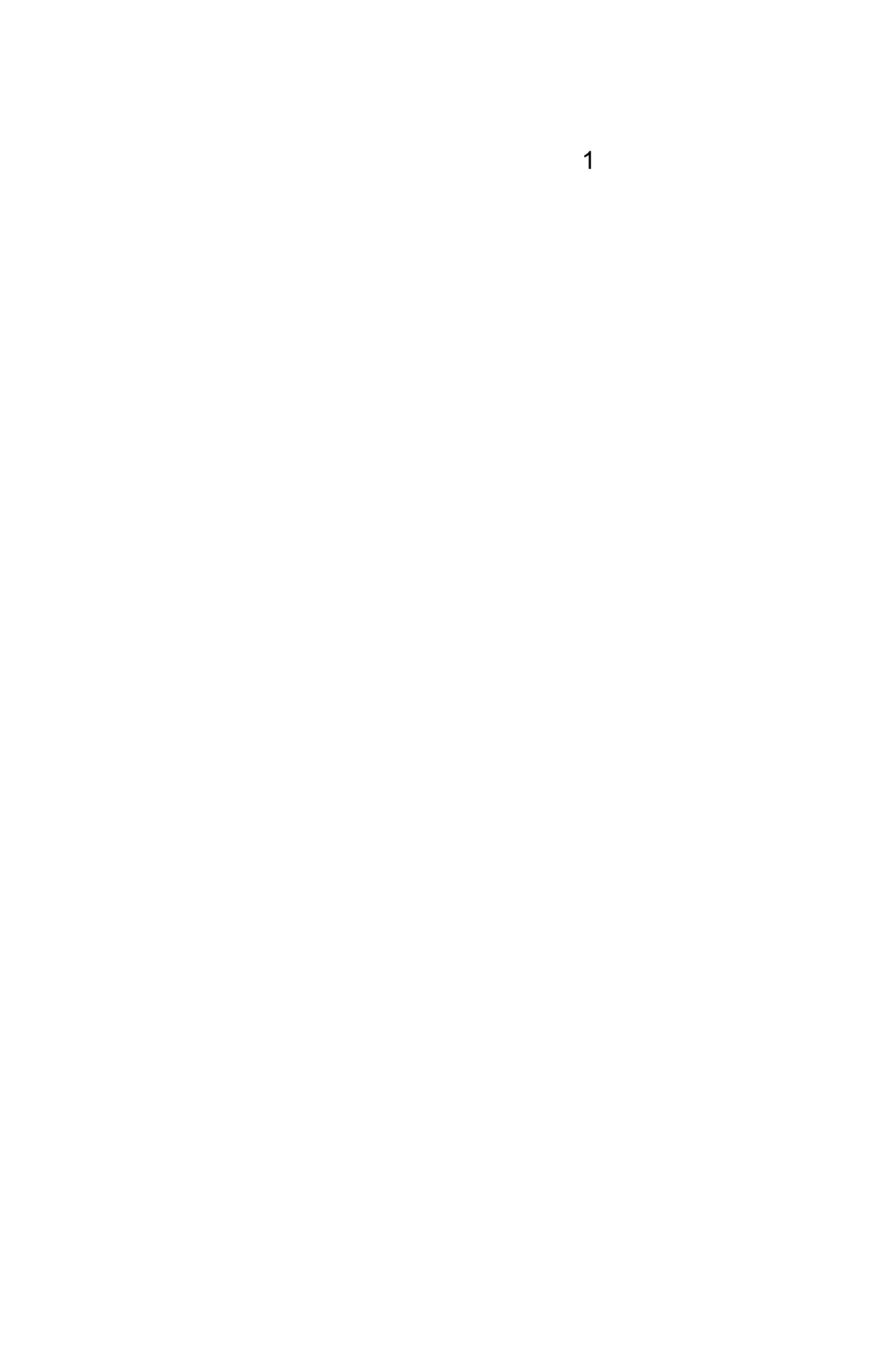


2
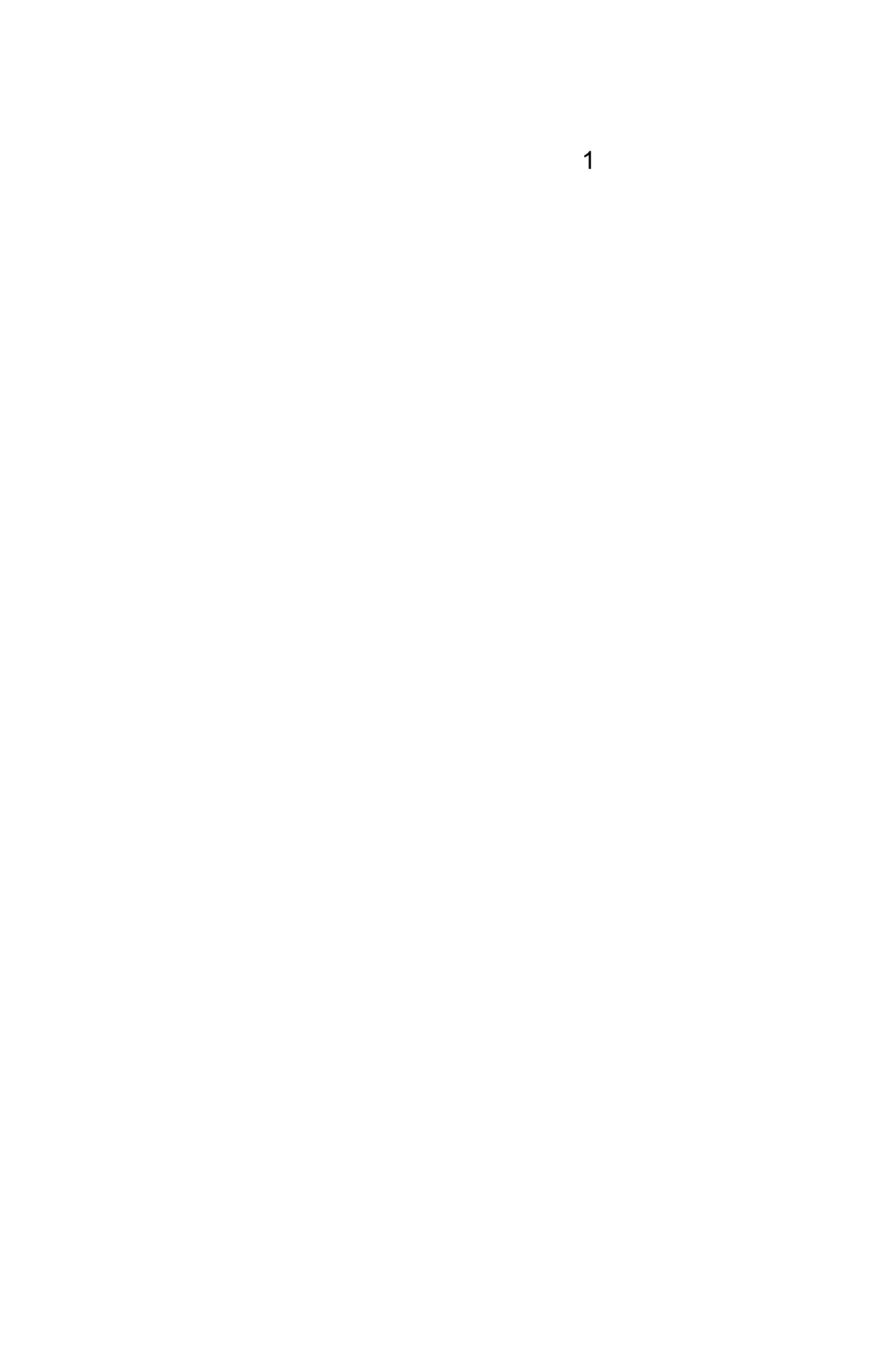


3
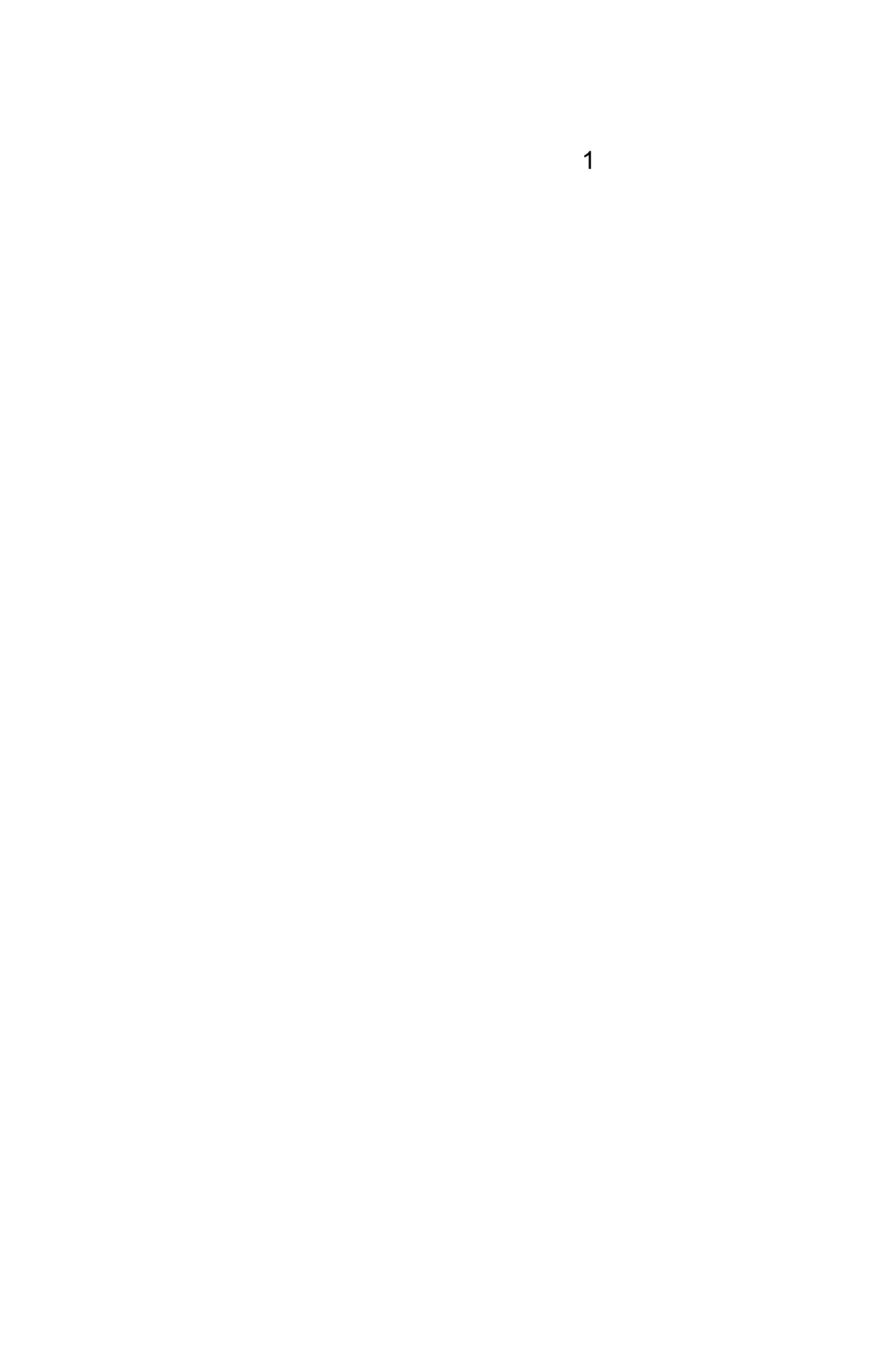


1

43

23

1

**
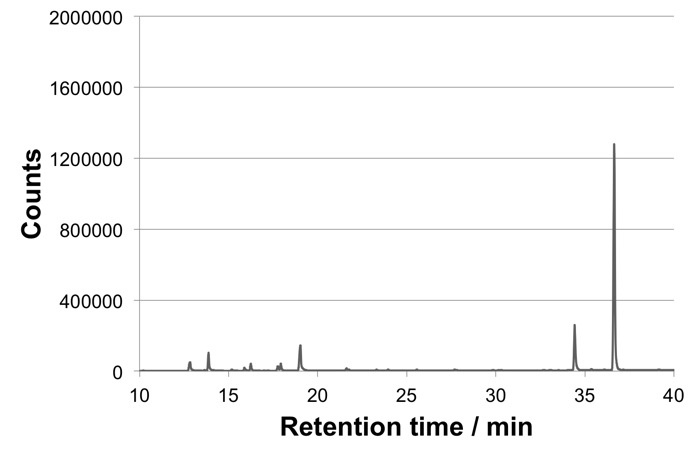

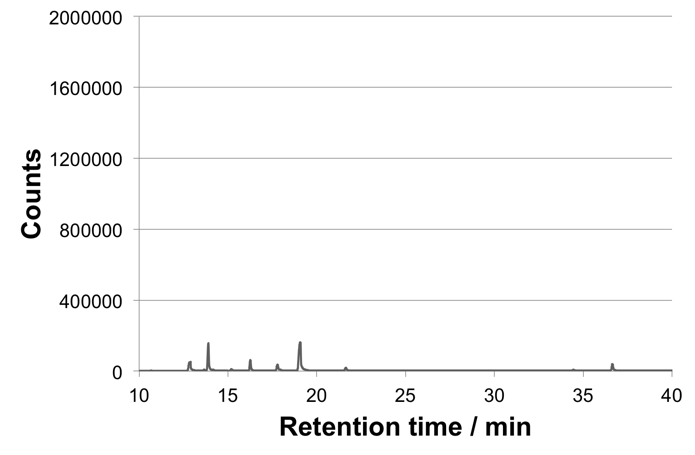
**

**OrfA (original)**

**OrfA 5×**

4

1

2

3

2

1

3

4

**
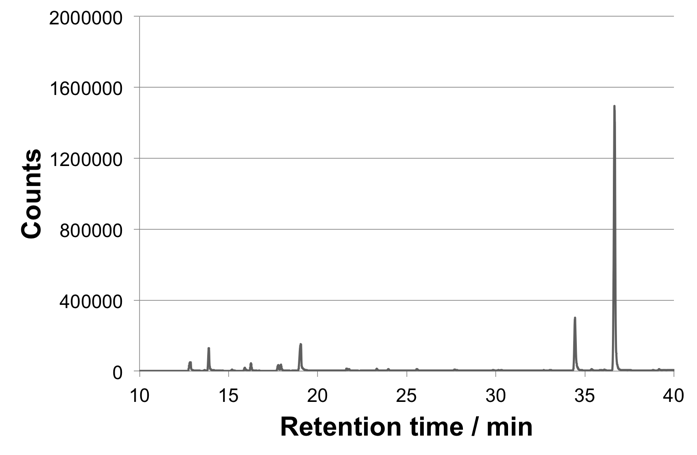

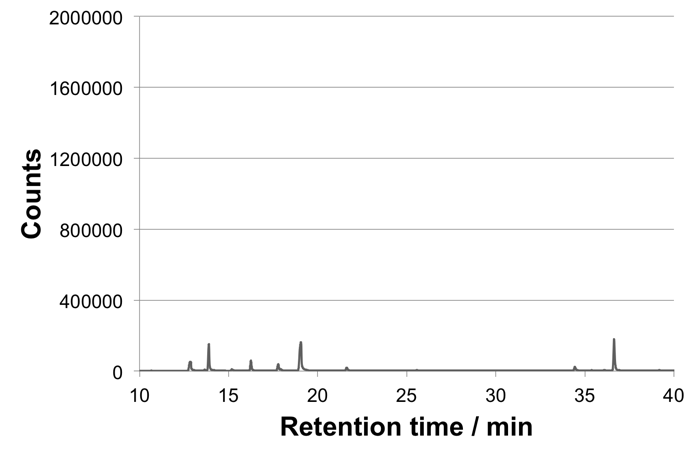
**

4

**OrfA 10×**

**OrfA 6×**

3

1

2

4

3

2

1

**
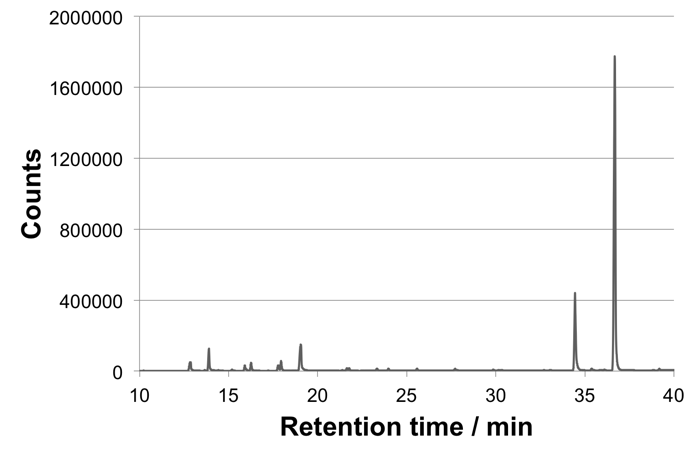

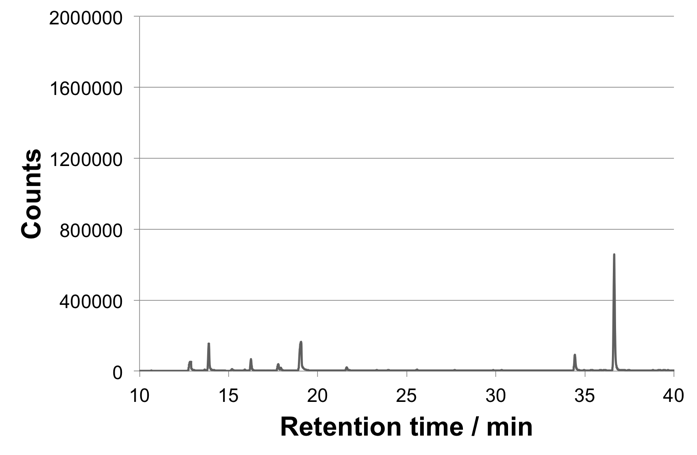
**

4

**OrfA 11×**

**OrfA 7×**

3

4

1

2

3

2

1

**(b)**

*m/z*

**4: DHA**

**3: DPAω6**

*m/z*

**Supplementary Fig. 3. GC/MS analysis of polyunsaturated fatty acids produced by OrfAs with 4× to 11×acyl carrier protein domains.** (a) Chromatograms traced at *m/z* 79. Polyunsaturated fatty acid methyl esters were identified by using authentic samples. DPAω6 and DHA were detected at 34.5 and 36.7 min, respectively. 1: C16:1ω9, 2: C18:1ω11, 3: DPAω6, 4: DHA. (b) Mass spectra of peak 3 and 4.


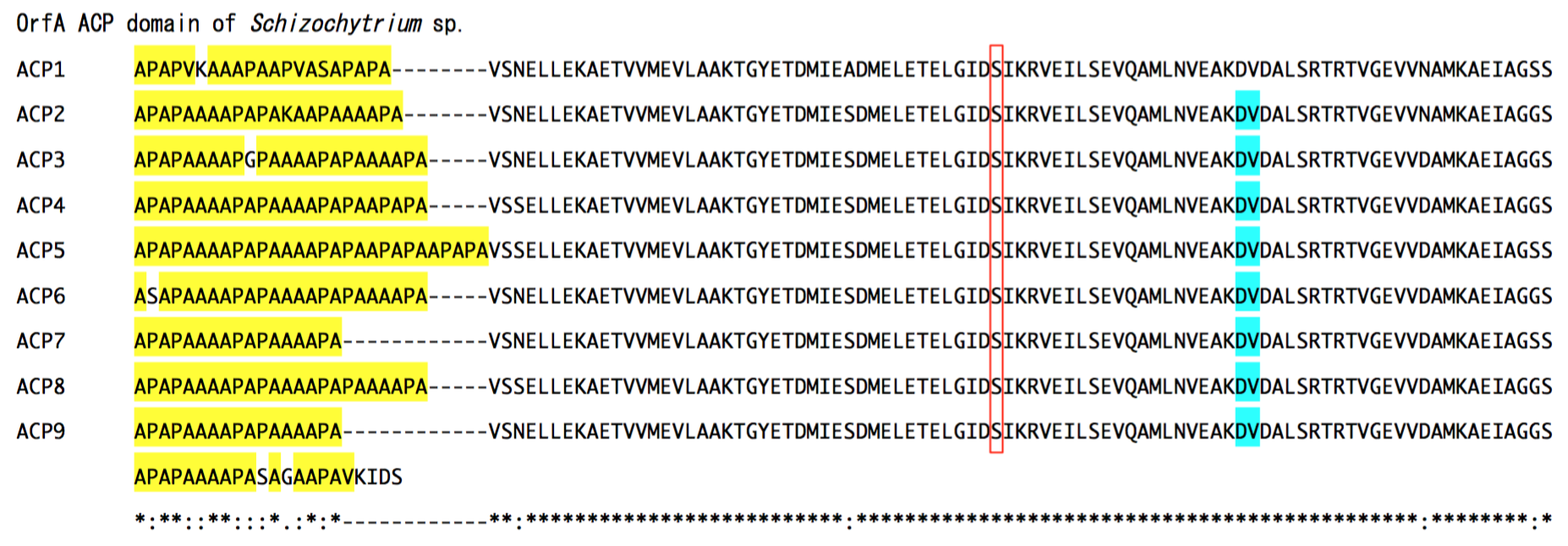


**Supplementary Fig. 4. Sequence alignment of the tandem acyl carrier protein domains of *orfA.*** The red box, yellow highlighting, and blue highlighting show the active sites of the ACP domains, Ala and Pro rich linkers, and *Zra*I sites, respectively.


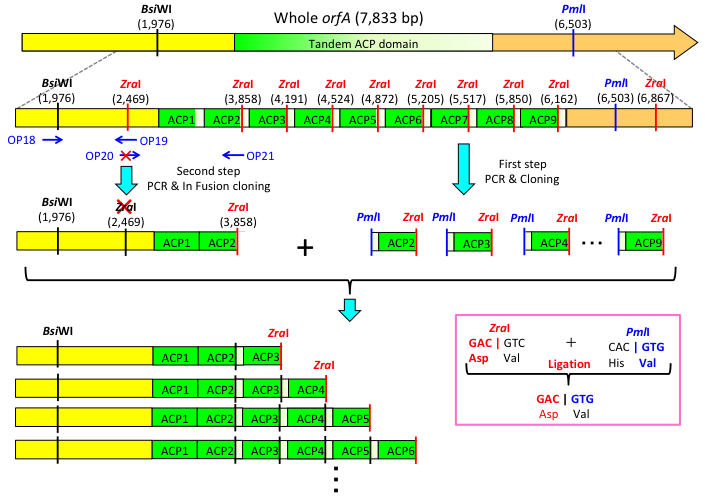

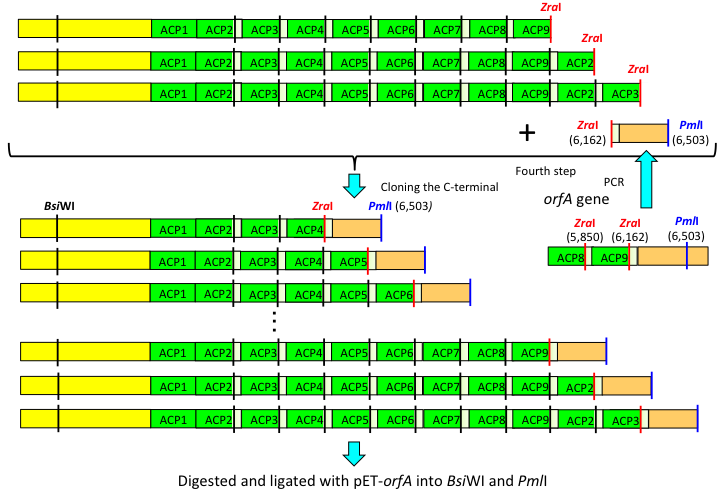


**Supplementary Fig. 5. Strategy for construction of *orfAs* with 4× to 11×acyl carrier protein domains.**


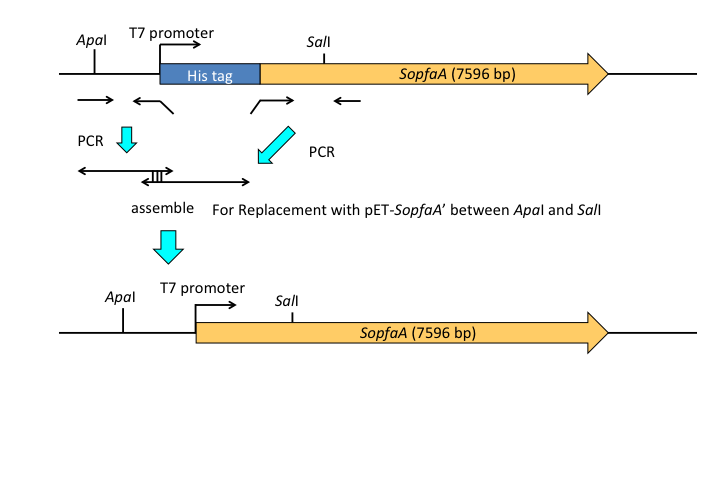


**Supplementary Fig. 6. Strategy for pET-*SopfaA* construction.**

**(a)**

**
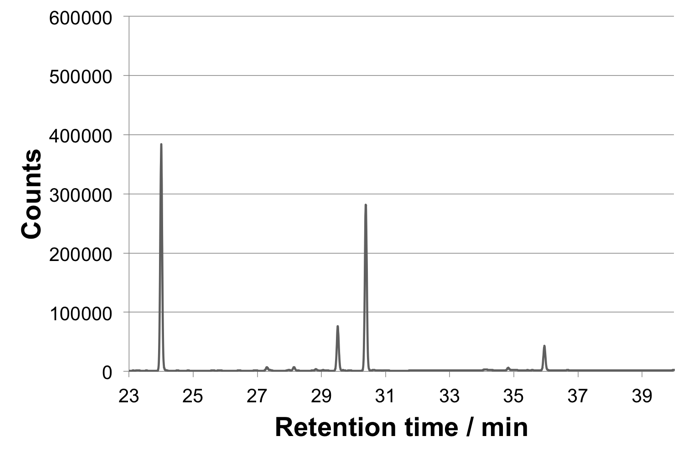

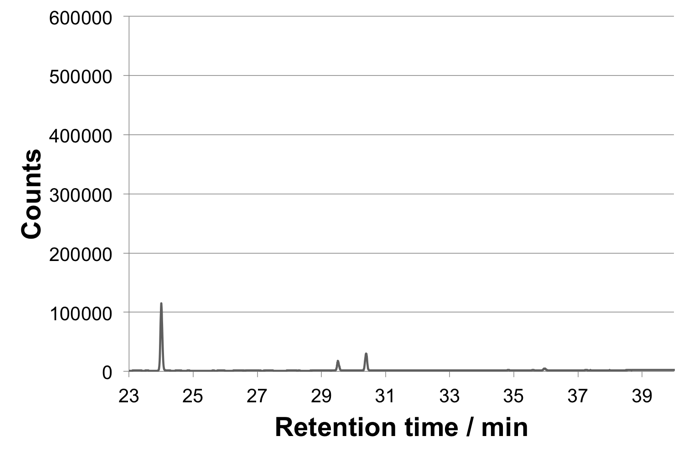
**

**SoPfaA 7×**

**SoPfaA (original)**

1

3

2

1

4

4

3

2

**
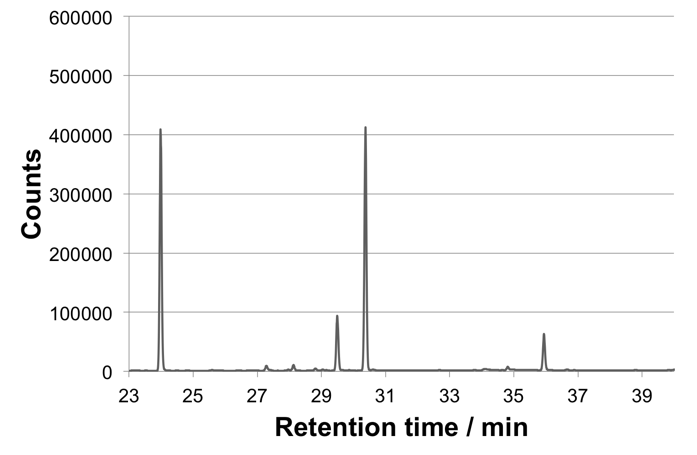

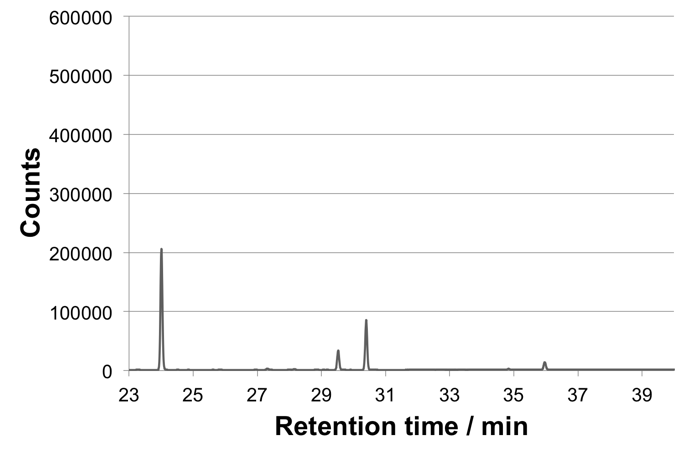
**

**SoPfaA 8×**

**SoPfaA 5×**

3

1

1

2

3

4

4

2

**
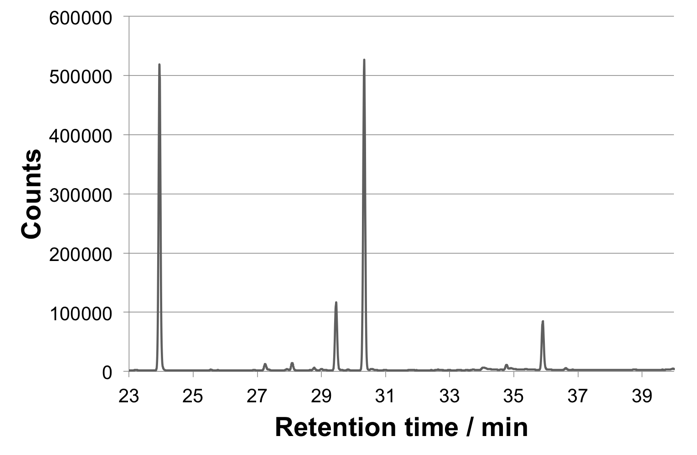

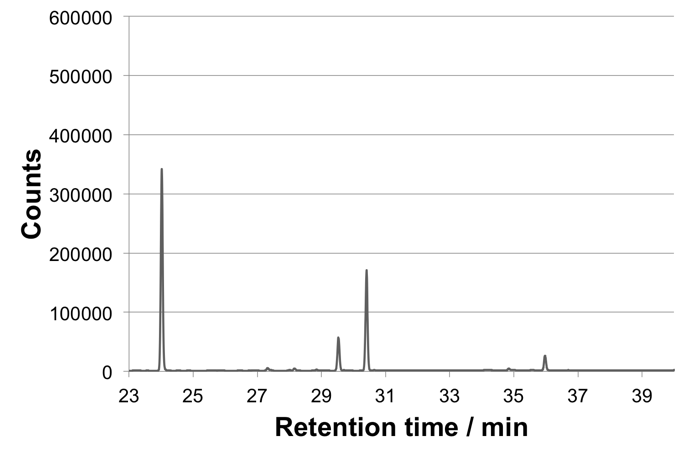
**

3

1

**SoPfaA 9×**

**SoPfaA 6×**

1

3

4

2

4

2

**(b)**

*m/z*

*m/z*

**2: ETA**

**1: SDA**

*m/z*

*m/z*

**4: DPAω3**

**3: EPA**

**Supplementary Fig. 7. GC/MS analysis of polyunsaturated fatty acids produced by SoPfaAs with 4× to 9× acyl carrier protein domains.** (a) Chromatograms traced at *m/z* 79. Polyunsaturated fatty acid methyl esters were identified by using authentic samples. SDA, ETA, EPA, and DPAω3 were detected at 24.0, 29.5, 30.4, and 36.0 min, respectively. 1: SDA, 2: ETA, 3: EPA, 4: DPAω3. (b) Mass spectra of peak 1, 2, 3, and 4.

**(a)**


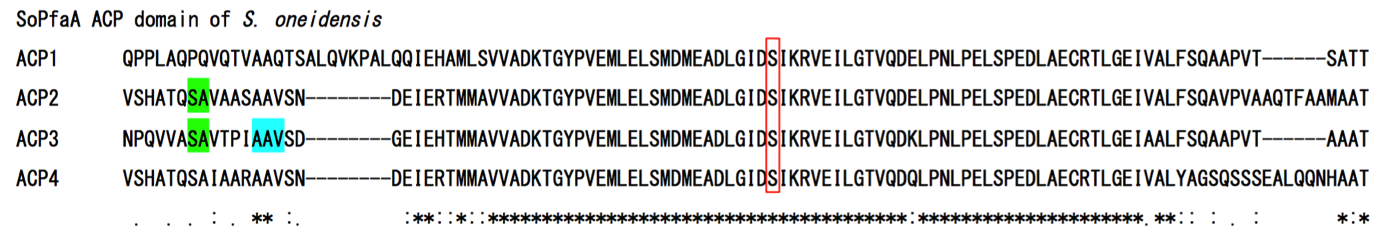


**(b)**


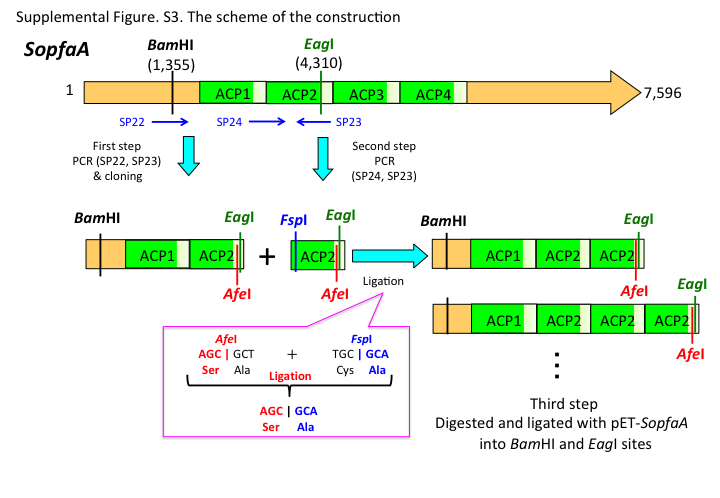


**Supplementary Fig. 8. Strategy for construction of *SopfaA* genes with 5× to 9×acyl carrier protein domains.**

(a) Sequence alignment of the tandem acyl carrier protein domains of *SopfaA*. The red box, blue highlighting, and green highlighting show the active sites of the ACP domains, the *Eag*I site, and the boundary of one unit of ACP, respectively.

(b) Plasmid construction is schematically shown.

**(a)**

**
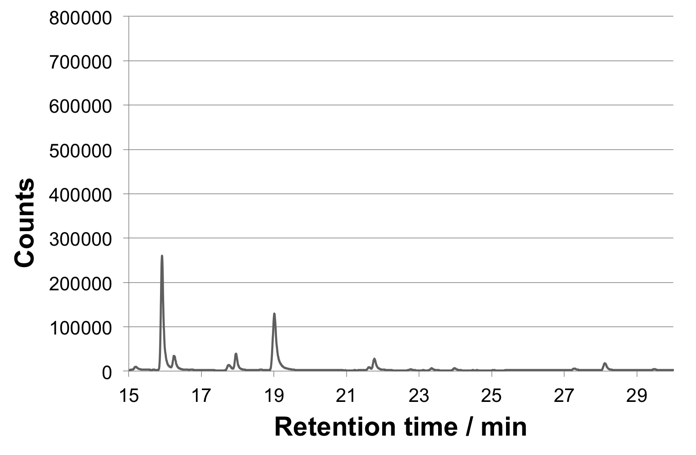

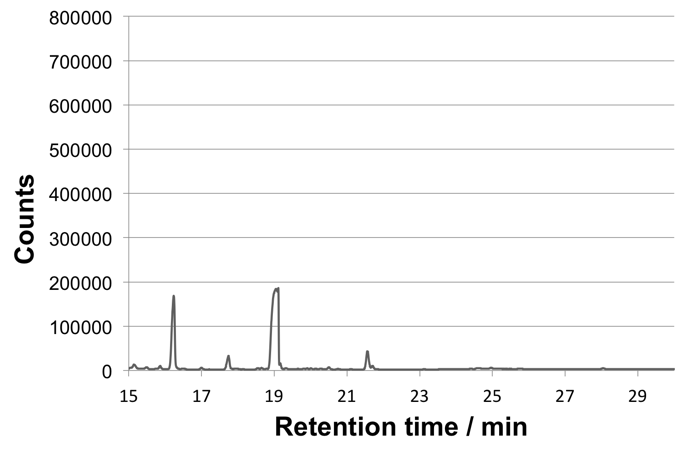
**

**SoPfaA**

**AmPfaA**

**
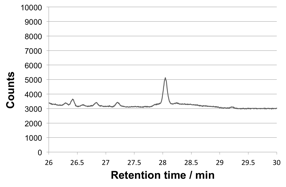
**

5

1

3

2

3

5

4

2


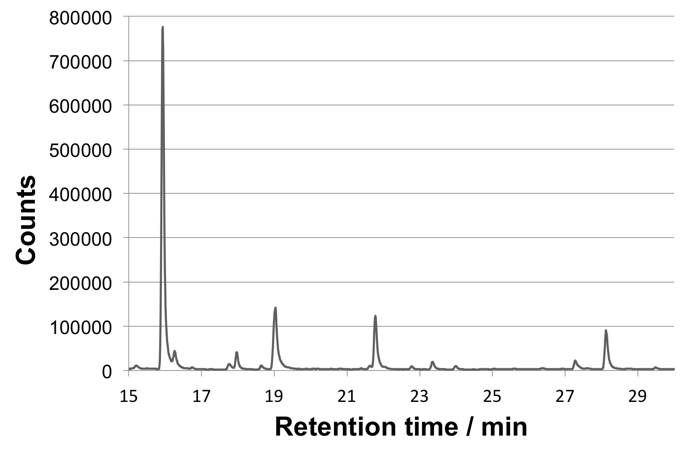
**
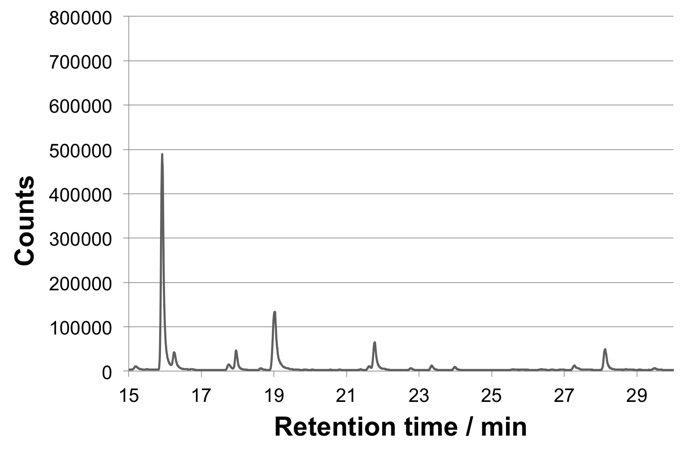
**

1

**SoPfaA 9**×

**SoPfaA 6**×

1

4

3

3

5

2

5

4

2

**(b)**

*m/z*

*m/z*

**4: GLA**

**1: HTA**

*m/z*

**5: ARA**

**Supplementary Fig. 9. GC/MS analysis of polyunsaturated fatty acids produced by engineered SoPfaAs and AmPfaB to E.** (a) Chromatograms traced at *m/z* 79. Polyunsaturated fatty acid methyl esters were identified by using authentic samples. GLA, and ARA were detected at 21.8 and 28.1 min, respectively. Peak 1 was identified as HTA by GC/MS analysis of the pyrrolidide derivative (Supplementary Fig. 10). 1: HTA, 2: C18:0, 3: C18:1ω11, 4: GLA, 5: ARA. (b) Mass spectra of peak 1, 4, and 5.

**
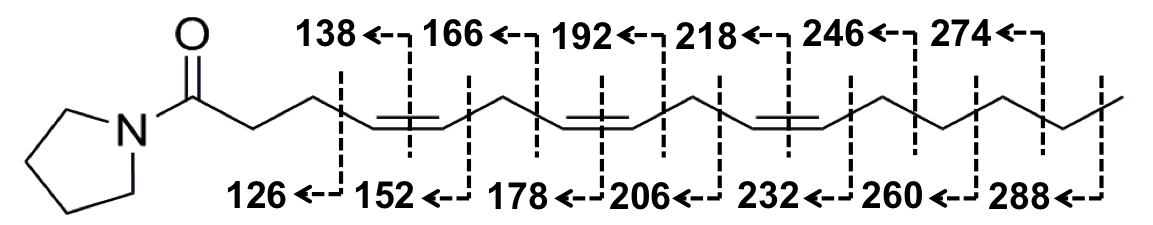
**

4×

*m/z*

**Supplementary Fig. 10. Mass spectrum of the pyrrolidide derivative of 4,7,10-hexadecatrienoic acid (HTA) produced by AmPfaA, SoPfaA, SoPfaA6, and SoPfaA9.** The estimated fragment pattern is shown schematically (upper panel).

**Supplementary Table 1.** Polyunsaturated fatty acids produced by polyunsaturated fatty acid synthase from *Schizochytrium* sp. with engineered *orfA*s.

Number of OD DPAω6 (ratio)^*^ DHA (ratio)^*^ Total PUFAs^**^ (ratio)^*^

ACP domains µg ml^−1^ OD^−1^ µg ml^−1^ OD^−1^ µg ml^−1^ OD^−1^

4 14.0 ± 0.3 not detected 0.018 ± 0.0009 (0.004) 0.018 ± 0.0009 (0.003)

5 14.5 ± 0.2 0.0092 ± 0.0009 (0.01) 0.11 ± 0.008 (0.03) 0.12 ± 0.008 (0.02)

6 14.6 ± 0.2 0.051 ± 0.002 (0.07) 0.52 ± 0.01 (0.11) 0.58 ± 0.01 (0.11)

7 13.9 ± 0.2 0.22 ± 0.002 (0.31) 1.9 ± 0.02 (0.42) 2.2 ± 0.02 (0.41)

8 14.0 ± 0.4 0.48 ± 0.004 (0.68) 3.7 ± 0.03 (0.83) 4.2 ± 0.03 (0.80)

9 (native) 14.3 ± 0.4 0.71 ± 0.03 4.5 ± 0.08 5.2 ± 0.1

10 14.0 ± 0.3 1.1 ± 0.04 (1.5) 6.6 ± 0.1 (1.5) 7.6 ± 0.2 (1.5)

11 14.4 ± 0.3 1.5 ± 0.02 (2.0) 8.0 ± 0.2 (1.8) 9.5 ± 0.2 (1.8)

^*^Relative values with the value of the native enzyme taken as 1.

^**^Sum of DPAω6 and DHA.

Data are presented as mean values ± standard deviation derived from three independent experiments.

**Supplementary Table 2.** Polyunsaturated fatty acids produced by polyunsaturated fatty acid synthase from *Shewanella oneidensis* with engineered *SopfaA*s.

Number of OD SDA (ratio)^*^ ETA (ratio)^*^ EPA (ratio)^*^ DPAω3 (ratio)^*^ Total PUFAs^**^ (ratio)^*^

ACP domains µg ml^−1^ OD^−1^ µg ml^−1^ OD^−1^ µg ml^−1^ OD^−1^ µg ml^−1^ OD^−1^ µg ml^−1^ OD^−1^

4 (native) 21.6 ± 1.5 0.22 ± 0.02 0.045 ± 0.003 0.10 ± 0.002 0.013 ± 0.003 0.38 ± 0.007

5 22.2 ± 0.9 0.46 ± 0.02 (2.1) 0.14 ± 0.004 (3.0) 0.38 ± 0.01 (3.7) 0.051 ± 0.002 (4.0) 1.0 ± 0.03 (2.7)

6 24.6 ± 0.8 0.62 ± 0.02 (2.8) 0.16 ± 0.005 (3.5) 0.52 ± 0.01 (5.1) 0.065 ± 0.004 (5.1) 1.4 ± 0.04 (3.6)

7 22.3 ± 1.2 0.92 ± 0.02 (4.1) 0.26 ± 0.005 (5.8) 1.1 ± 0.05 (10) 0.13 ± 0.006 (9.7) 2.4 ± 0.06 (6.2)

8 21.4 ± 1.4 1.1 ± 0.08 (4.8) 0.34 ± 0.02 (7.5) 1.6 ± 0.06 (16) 0.18 ± 0.006 (14) 3.1 ± 0.1 (8.2)

9 21.5 ± 1.3 0.94 ± 0.08 (4.2) 0.33 ± 0.02 (7.2) 1.6 ± 0.08 (16) 0.19 ± 0.01 (15) 3.0 ± 0.2 (8.0)

^*^Relative values with the value of the native enzyme taken as 1.

^**^Sum of SDA, ETA, EPA, and DPAω3.

Data are presented as mean values ± standard deviation derived from four independent experiments.

**Supplementary Table 3.** Constructs with SoPfaA derivatives.

name Description identity^*^ (amino acid length)

SoPfaA5 active 2^nd^ ACP domain of SoPfaA was inserted between the 2^nd^ and

3^rd^ ACP domains of SoPfaA. (115)

SoPfaA5-3M inactive 2^nd^ ACP domain of SoPfaA was inserted between the 2^nd^ and

3^rd^ ACP domains of SoPfaA. (115)

SoPfaA-MmPfaA-M inactive 2^nd^ ACP domain of MmPfaA was inserted between the 2^nd^ and

3^rd^ ACP domains of SoPfaA. 70% (103)

SoPfaA-PpPfaA-M inactive 2^nd^ ACP domain of PpPfaA was inserted between the 2^nd^ and

3^rd^ ACP domains of SoPfaA. 71% (107)

SoPfaA-OrfA-M inactive 3^rd^ ACP domain of OrfA was inserted between the 2^nd^ and

3^rd^ ACP domains of SoPfaA. 49% (109)

SoPfaA-AmPfaA-M inactive 2^nd^ ACP domain of AmPfaA was inserted between the 2^nd^ and

3^rd^ ACP domains of SoPfaA. 48% (107)

SoPfaA-S1 118^th^ to 224^th^ amino acid of HlyB protein of *S. oneidensis* was inserted

between the 2^nd^ and 3^rd^ ACP domains of SoPfaA. (107)

SoPfaA-S2 383^th^ to 489^th^ amino acid of HlyB protein of *S. oneidensis* was inserted

between the 2^nd^ and 3^rd^ ACP domains of SoPfaA. (107)

^*^Compared with the 2^nd^ acyl carrier protein (ACP) domain of SoPfaA.

**Supplementary Table 4.** Amino acid sequences of S1 and S2 of the HlyB protein of *Shewanella oneidensis.*

Name Amino acid sequence

S1 LPSLNQKRSRFASQFKQQKKWFKPVFLLSLVASLTGLAIPLFTMAVYDRVIG

GQAPNILPGIAIGAFLALSIFISSRLLRAKVLASASNKLARDLSAVSFNQLLSMP

S2 LNQSISPGALIACGMLIWRITGPAQLAFSSAPKINMINSTVTQFDRFMEVTTEF

NQLRLDGPNLNKAPALNFKHVTLRYTAEAEPALSGVTFDVEAGETVAIIGPNG

**Supplementary Table 5.** Polyunsaturated fatty acids produced by engineered *SopfaA* and *AmpfaB* to *E.*

The subunit A OD HTA (ratio)^*^ GLA (ratio)^*^ ARA (ratio)^*^ Total PUFAs^**^ (ratio)^*^

µg ml^−1^ OD^−1^ µg ml^−1^ OD^−1^ µg ml^−1^ OD^−1^ µg ml^−1^ OD^−1^

AmPfaA 19.7 ± 0.8 0.00088 ± 0.00008 0.0012 ± 0.0002 0.00044 ± 0.00009 0.0024 ± 0.0005

SoPfaA 20.5 ± 0.3 0.16 ± 0.01 0.044 ± 0.0004 0.021 ± 0.0006 0.22 ± 0.01

SoPfaA6 20.3 ± 0.2 0.29 ± 0.01 (1.8) 0.098 ± 0.002 (2.2) 0.058 ± 0.001 (2.8) 0.45 ± 0.02 (2.0)

SoPfaA9 19.8 ± 0.4 0.47 ± 0.0 (3.0) 0.18 ± 0.008 (4.2) 0.11 ± 0.005 (5.4) 0.77 ± 0.02 (3.4)

^*^Relative values with the value of the native SoPfaA taken as 1.

^**^Sum of HTA, GLA, and ARA.

Data are presented as mean values ± standard deviation derived from four independent experiments.

HTA values were estimated with a GLA calibration curve.

**Supplementary Table 6.** Strains used in this study.

Strains Description (accession number) Source

*E. coli* XL1-Blue hsdR17, rec*A*1, end*A*1, gyr*A*96, thi-1, supE44, rel*A*1, Nippon Gene

lac[F’, pro*AB*, lacI^q^Z*∆*M15, Tn10(tet^R^)]

*E. coli* BLR(DE3) F^−^, *ompT hsdS*_B_(r_B_^−^ m_B_^−^) *gal dcm* (DE3) Δ(*srl-recA*)306::Tn*10* (*tet*^R^) Merck

*E. coli* BLR(DE3) *∆fadE* BLR(DE3) derivative, *∆fadE* This study

*Schizochytrium* sp. *orfABC* (AF378327, AF378328, AF378329) ATCC 20888

*Shewanella oneidensis* MR-1 *SopfaABCDE* (NC_004347) ATCC BAA-1096

*Aureispira marina* *AmpfaABCDE* (AB980240) JCM23201

*Photobacterium profundum* SS9 *PppfaA* (CR354531) ATCC BAA-1252

*Moritella marina* MP-1 *MmpfaA* (AB025342) ATCC 15381

*Nostoc* sp. PCC 7120 *hetI* (L22883) ATCC27893

ATCC; American Type Culture Collection, JCM; Japan Collection of Microorganisms RIKEN Bioresource Center.

**Supplementary methods**

**1. Deletion of the *fadE* gene in *Escherichia coli* BLR(DE3)**

To construct a *fadE* disruptant, the Quick & Easy *Escherichia coli* Gene Deletion Kit (Gene Bridges GmbH, Heidelberg, Germany) was used according to the manufacturer’s protocol (Supplementary Fig. 2). In brief, DNA fragments possessing a Km-resistance gene cassette flanked with FRT sites and 50-bp homologous arms whose sequences were identical to the target regions were amplified by PCR with primers (KO01/KO02). The amplified DNA fragments were used to transform *E. coli* BLR(DE3) (Merck KGaA, Darmstadt, Germany). Gene disruption in the Km-resistant colonies was confirmed by PCR using appropriate sets of primers that hybridized approximately 300 bp upstream and downstream of the target genes (Supplementary Table 3). The sequences of the amplicons were then analysed to confirm the deletion. After that, the selection marker in the obtained mutant was removed with FLP-recombinase and the gene deletion was confirmed by PCR and direct sequencing of the amplicons.

**2. Plasmid constructs for expressing the *orfABC* and *hetI* genes**

Plasmid construction of pET-*orfA*

The *orfA* gene (8,733 bp; accession number AF378327) of PUFA synthase from *Schizochytrium* sp. ATCC20888 was amplified by PCR with primers (OP01/OP02) and genomic DNA of *Schizochytrium* sp. according to the manufacturer’s protocol. The amplified DNA fragment was digested with *Nde*I and *Eco*RI, and inserted into the corresponding sites of pET-21a (Merck).

Plasmid construction of pCDF-*orfB*

Previously, Metz *et al*. succeeded in expressing *orfB* with altered Ser codons suitable for *E. coli* though *orfB* encoded by the native codons was hardly expressed in *E. coli*^1^. Therefore, we constructed a plasmid carrying the *orfB* gene (6,180 bp; accession number AF378328) with the same altered Ser codons. The scheme is shown in Supplementary Fig. 1. First, DNA fragment 1 (4,568 bp to end of *orfB*) was amplified by PCR with primers (OP03/OP04) and genomic DNA of *Schizochytrium* sp. as a template. A pCDF-1b (Merck) derivative possessing *Nde*I and *Eco*RI sites was also constructed by PCR with two primers (OP05/OP06) and pCDF-1b as a template. The amplified fragment 1 was inserted into the *Nde*I and *Eco*RI sites of the pCDF derivative vector to obtain pCDF-*orfB1*.

Second, DNA fragment 2 (4,123 to 4,705 bp), which has the same altered Ser codons as those reported previously, was purchased from Thermo Fisher Scientific Inc. (Waltham, MA, USA). DNA fragment 3 (3,390 to 4,151 bp) was obtained by PCR with primers (OP07/OP08) and genomic DNA. To obtain DNA fragment 4 (3,390 to 4,705 bp), DNA fragments 2 and 3 were assembled by overlap extension PCR with primers (OP07/OP09). Fragment 4 was digested with *Nde*I and *Sac*II, and then inserted into the corresponding sites of pCDF-*orfB1* to construct pCDF-*orfB4*.

Finally, DNA fragment 5 (1 to 3,478 bp) was amplified by PCR with primers (OP10/OP11), digested with *Nde*I and *Mfe*I, and inserted into the same sites of pCDF-*orfB4* to yield pCDF-*orfB*.

Plasmid construction of pCOLA-*orfC*

The *orfC* gene (4,509 bp; accession number AF378329) was amplified by PCR with primers (OP12/OP13) and *Schizochytrium* sp. genomic DNA as a template. The fragment was digested with *Nde*I and *Eco*RI, and then inserted into the *Nde*I and *Mfe*I sites of pCOLADuet-1 vector (Merck).

Plasmid construction of pSTV-*hetI*

A 4ʹ-phosphopantetheinyl transferase (PPTase) is required to activate acyl carrier proteins (ACPs). Though no genes encoding PPTase have been reported from *Schizochytrium* sp., Metz *et al*. successfully used a PPTase gene, *het*I, from *Nostoc* sp. as an alternative^1^. We therefore used the *hetI* gene (accession number L22883), the codons of which were optimized for *E. coli* expression (Thermo Fisher Scientific). The DNA fragment was digested with *Nde*I and *Eco*RI and then inserted into the same sites of pSTV28N, which is a derivative of the pSTV28 vector (Takara Bio Inc., Shiga, Japan) and was constructed by inverse PCR with pSTV28 as a template and primers (OP14/OP15), to create an *Nde*I site at the start codon for protein expression. The plasmid thus obtained was designated pSTV-*hetI*.

**3. Plasmid constructs for expressing the *SopfaABCDE* genes**

Plasmid construction of pET-*SopfaA*

The *SopfaA* gene (7,596 bp; accession number NC_004347) of PUFA synthase from *Shewanella oneidensis* MR-1 was amplified by PCR with primers (SP01/SP02) and *S. oneidensis* MR-1 genomic DNA as a template (Supplementary Fig. 6). The fragment was digested with *Eco*RI and *Xho*I, and inserted into the same sites of pET-21a (Merck) to obtain pET-*SopfaA*ʹ. Then, the *N*-terminal His-tag sequences of the plasmid were deleted by overlap PCR extension with two sets of primers (SP03/SP04 and SP05/SP06) and pET-*SopfaA*ʹ as a template. Thus, the DNA region between the *Apa*I and *Sal*I sites in pET-*SopfaA*ʹ was replaced with the assembled DNA fragment to yield pET-*SopfaA*.

Plasmid construction of pCDF-*SopfaC*

The *SopfaC* gene (5,892 bp; accession number NC_004347) was amplified by PCR with primers (SP07/SP08) and genomic DNA as a template. The fragment was digested with *Pml*I and *Xho*I, and inserted into the same sites of pCDF-1b (Merck) to make pCDF-*SopfaC*ʹ. The *N*-terminal His-tag sequences of the plasmid were deleted as described above with primers (SP03/SP09 and SP10/SP11).

Plasmid construction of pCOLA-*SopfaD*

The *SopfaD* gene (1,644 bp; accession number NC_004347) was amplified by PCR with primers (SP12/SP13) and genomic DNA as a template. The fragment was digested with *Nde*I and *Bam*HI, and inserted into the *Nde*I and *Bgl*II sites of pCOLADuet-1.

Plasmid construction of pACYC-*SopfaEB*

For co-expression of *SopfaB* and *SopfaE* (accession number NC_004347), pACYCtrm, a derivative of pACYCDuet-1 (Merck), was constructed by inserting the *rrnB* terminator from pTrc99A^2^ between the first multi-cloning site and the second T7 promoter in pACYCDuet-1. Two DNA fragments possessing *rrnB* terminators and the second T7 promoter were prepared by PCR with primers (SP18/SP19 and SP20/SP21) and pACYCDuet-1 and pTrc99A as templates, and then assembled by overlap extension PCR. The DNA fragment thus obtained was digested with *Not*I and *Nde*I, and inserted into the same sites of pACYCDuet-1 to construct pACYCtrm*.*

The *SopfaE* gene was amplified by PCR with primers (SP14/SP15) and genomic DNA as a template, and then the *Nco*I and *Bam*HI fragment was inserted into the same sites of pACYCtrm to obtain pACYC-*SopfaE*. Then, the *SopfaB* gene was amplified by PCR with primers (SP16/SP17) and genomic DNA as a template, digested with *Nde*I and *Bam*HI, and inserted into the *Nde*I and *Bgl*II sites of pACYC-*SopfaE* to construct pACYC-*SopfaEB.*

**4. Plasmid constructs for expressing the *AmpfaABCDE* genes**

Plasmid construction of pET-*AmpfaA*

The *AmpfaA* gene (4,671 bp; accession number AB980240) of PUFA synthase from *Aureispira marina* was amplified by PCR with primers (AP01/AP02) and pSTV29-*Plac-pfaAB*^3^ as a template. The fragment was digested with *Nde*I and *Bam*HI, and inserted into the same sites of pET-21a.

Plasmid construction of pCDF-*AmpfaC*

The *AmpfaC* gene (6,726 bp; accession number AB980240) was amplified with primers (AP03/AP04) and pMW219-*Plac-pfaCD*^3^ as a template. The fragment digested with *Nde*I and *Bam*HI was replaced with that of pCDF-*orfB.*

Plasmid construction of pCOLA-*AmpfaD*

The *AmpfaD* gene (1,629 bp; accession number AB980240) was amplified with primers (AP05/AP06) and pMW219-*Plac-pfaCD*^3^ as a template. The *Nde*I and *Bam*HI digested fragment was inserted into the *Nde*I and *Bgl*II sites of pACYCDuet-1.

Plasmid construction of pACYC-*AmpfaEB* and pACYC-*SopfaE*-*AmpfaB*

The *AmpfaE* gene (660 bp; accession number AB980240) and *AmpfaB* gene (2,406 bp; accession number AB980240) were each amplified with primers (AP07/AP08 and AP09/AP10, respectively) and template DNA (pUC19-*Plac-pfaE* and pSTV29-*Plac-pfaAB*^3^, respectively). The former and latter fragments were digested with *Nco*I/*Bam*HI and *Nde*I/*Bam*HI, respectively, and inserted into the *Nco*I/*Bam*HI and *Nde*I/*Bgl*II sites of pACYCtrm to construct pACYC-*AmpfaEB*.

To construct pACYC-*SopfaE-AmpfaB*, pACYC-*AmpfaEB* was digested with *Nde*I and *Xho*I, and inserted into the same sites of pACYC-*SopfaE*.

**5. Construction of *orfA* genes with 4 to 11 acyl carrier protein domains**

To investigate the relationship between PUFA productivity and the number of ACP domains in the *orfA* gene, we constructed *orfA* genes possessing 4×, 5×, 6×, 7×, 8×, 9×, 10×, and 11×ACP domains. Each of the ACP domains is highly conserved (Supplementary Fig. 4) and separated by conserved and repeated regions with Ala and Pro rich sequences (Supplementary Fig. 4, yellow). We therefore considered the region between Ala/Pro rich sequences one ACP domain unit.

To construct the plasmids, we used the following technique. *Zra*I sites exist in every ACP domain except the first one (Supplementary Fig. 5). A *Pml*I site is also located downstream of the last ACP domain. Deletion of the internal region between either *Zra*I or *Pml*I in the *orfA* gene results in no frame shift of codons. Moreover, the DNA fragment obtained by ligation at the *Zra*I and *Pml*I sites cannot be re-digested with either of the restriction enzymes.

First, each of the single ACP domains was randomly amplified by PCR with primers (OP16/OP17) using pET-*orfA* as a template. The fragment was digested *Hin*dIII/*Pst*I and inserted into the same sites of pUC18 (Takara Bio). By sequencing, we identified the original ACP domain and consequently obtained plasmids possessing each of the ACP domains (pUC18-ACP2^nd^, 3^rd^, 4^th^, 5^th^, 6^th^, 7^th^, 8^th^ and 9^th^).

Next, an *orfA* gene fragment from 1,976 to 2,469 bp was amplified with pET-*orfA* as a template and primers (OP18/OP19); the primers were designed to remove the additional *Zra*I site located at 2,469 bp. An *orfA* gene fragment from 2,469 bp to 3,858 bp was amplified with primers (OP20/OP21) and pET-*orfA* as a template. A pHSG298 (Takara Bio) derivative plasmid possessing *Bsi*WI and *Zra*I sites was also constructed by inverse PCR with primers (OP22/OP23) and pHSG298 as a template. The three fragments thus obtained were assembled by in-fusion recombination (Takara Bio) to obtain pHSG298-ACP2ʹ.

Next, we obtained the ACP fragment by digesting pUC18-ACP3 with *Pml*I and *Bam*HI. The fragment was inserted into the *Zra*I and *Bam*HI sites of pHSG298-ACP2ʹ. This operation was repeated to construct plasmids possessing the desired number of ACP domains (pHSG298-4ʹ to 11ʹ). Then, an *orfA* gene fragment from 6,162 to 6,503 bp was amplified with primers (OP24/OP25) and pET-*orfA* as a template. The fragment was digested with *Zra*I and *Bam*HI, and inserted into the same sites of pHSG298-ACP4ʹ to 11ʹ to construct pHSG298-ACP4 to 11. Finally, the *orfA* genes carrying the desired number of ACP domains were digested with *Bsi*WI and *Pml*I, and replaced with those of pET-*orfA* to make pET-*orfA4* to *11*, respectively.

**6. Plasmid construction of *SopfaA* genes with 5 to 9 acyl carrier protein domains**

We also constructed *SopfaA* genes possessing 5×, 6×, 7×, 8×, and, 9×ACP domains. In this case, we employed the same technical strategy as that used to construct *orfA* genes with 4 to 11 ACP domains (Supplementary Fig. 8).

First, a fragment from the *Bam*HI to *Eag*I site was amplified by PCR with pET-*SopfaA* as a template and primers (SP22/SP23); the primers were designed to create an *Afe*I site in the Ser1431 and Ala1432 codons. The fragment was digested with *Bam*HI and *Eco*RI, and inserted into the same sites of pHSG298 to obtain pHSG298-ACP2. Then, a fragment carrying the second ACP domain was amplified with primers (SP24/SP23) and pET-*SopfaA* as a template. In this case, the primer SP24 was designed to create a *Fsp*I site in the Ser1323 and Ala1324 codons. The fragment was digested with *Fsp*I and *Eco*RI, and inserted into the *Afe*I and *Eco*RI sites of pHSG298-ACP2 to obtain pHSG298-ACP3. This operation was repeated to construct plasmids with the desired number of ACP domains (pHSG298-ACP3 to 7). Finally, the *SopfaA* genes possessing the desired number of ACP domains were digested with *Bam*HI and *Eag*I, and replaced with the corresponding region of pET-*SopfaA* to construct pET-*SopfaA5* to *9.*

1. **Plasmid constructs with inactivated or modified ACP domains.**

Plasmid construction of pET-*SopfaA5-1M*

A DNA fragment, in which the first ACP was inactivated by replacing the active Ser with an Ala residue, was amplified by overlap PCR with primers (SP28/SP29/SP25/SP26) and pHSG298-ACP2 as a template. The fragment was digested with *Nde*I and *Eco*RI, and replaced with the corresponding region of pHSG298-ACP2 to obtain pHSG298-ACP2-1M. A DNA fragment with active ACP domains was amplified with primers (SP24/SP23) and pHSG298-ACP2 as a template, digested with *Fsp*I and *Eco*RI, and inserted into the *Afe*I and *Eco*RI sites of pHSG298-ACP2-1M. The *Eag*I and *Bam*HI fragment of the plasmid thus obtained was replaced with that of pET-*SopfaA.*

Plasmid construction of pET-*SopfaA5-2M*

A DNA fragment, in which the second ACP was inactivated, was obtained as described above with primers (SP30/SP31/SP25/SP26) and pHSG298-ACP2 as a template. The fragment was digested with *Nde*I and *Eco*RI, and replaced with the corresponding fragment of pHSG298-ACP2 to construct pHSG298-ACP2-2M. After this, the protocol for pET-*SopfaA5-1M* construction was employed; that is, the preparation of a DNA fragment with active ACP domains by PCR with primers (SP24/SP23), the cloning of the *Fsp*I and *Eco*RI fragment into pHSG298-ACP2-2M, and the replacement of the *Eag*I and *Bam*HI fragments.

Plasmid construction of pET-*SopfaA5-3M*

A DNA fragment, in which the third ACP was inactivated, was obtained as described above with primers (SP24/SP23/SP30/SP31) and pHSG298-ACP2 as a template. The fragment was digested with *Fsp*I and *Eco*RI, and replaced with the *Afe*I and *Eco*RI fragment of pHSG298-ACP2 to make pHSG298-ACP3-3M. After this, the protocol described above was employed.

Plasmid construction of pET-*SopfaA5-4M*

The *Eag*I and *Xho*I fragment possessing the *C*-terminal half region was inserted into the same sites of the pBluescript II SK(+) vector (Agilent Technologies Inc., Santa Clara, CA, USA) to make pBlue-ACP34. A DNA fragment, in which the fourth ACP was inactivated, was obtained as described above with primers (SP32/SP33/SP27/AP02) and pBlue-ACP34 as a template. The *Eag*I and *Sac*I fragment of the constructed plasmid was replaced with that of pBlue-ACP34 to obtain pBlue-ACP3M4. Finally, the *Eag*I and *SacI* fragment of pET-*SopfaA* was replaced with that of pBlue-ACP3M4*.*

Plasmid construction of pET-*SopfaA5-5M*

A DNA fragment, in which the fifth ACP was inactivated, was obtained as described above with primers (SP34/SP35/SP27/AP02) and pBlue-ACP34 as a template. The fragment was digested with *Eag*I and *Sac*I, and replaced with that of pBlue-ACP34 to make pBlue-ACP34M. Then, the *Eag*I and *Sac*I fragment of pET-*SopfaA* was replaced with that of pBlue-ACP34M*.*

Plasmid construction of pET-*SopfaA-PppfaA-M*

A DNA fragment carrying the second mutated ACP domain of *PppfaA* (accession number CR354531) from *Photobacterium* *profundum* SS9 was amplified by overlap PCR with primers (SP36/SP37/SP38/SP39) and the *P. profundum* SS9 genome as a template. The fragment was digested with *Afe*I and *Eag*I, and inserted into the same sites of pHSG298-ACP2. Then, the *Eag*I and *Bam*HI fragment of the constructed plasmid was replaced with that of pET-*SopfaA.*

Plasmid construction of pET-*SopfaA-AmpfaA-M*

A DNA fragment carrying the second mutated ACP domain of *AmpfaA* from *A. marina* was amplified by overlap PCR with primers (SP40/SP41/SP42/SP43) and pET-*AmpfaA* as a template. After this, the method for pET-*SopfaA-PppfaA-M* construction was employed*.*

Plasmid construction of pET-*SopfaA-MmpfaA-M*

A DNA fragment carrying the second mutated ACP domain of *MmpfaA* (accession number AB025342) from *Moritella marina* was amplified by overlap PCR with primers (SP44/SP45/SP46/SP47) and the *M. marina* genome as a template. The fragment was digested with *Fsp*I and *Eag*I, and inserted into the *Afe*I and *Eag*I sites of pHSG298-ACP2. The replacement of the *Eag*I and *Bam*HI fragments was carried out using the method described above*.*

Plasmid construction of pET-*SopfaA-orfA-M*

A DNA fragment carrying the third mutated ACP domain of *orfA* from *Schizochytrium* sp. was amplified by overlap PCR with primers (SP48/SP49/SP50/SP51) and pET-*orfA* as a template. The fragment was digested with *Fsp*I and *Eag*I, and inserted into the *Afe*I and *Eag*I sites of pHSG298-ACP2. The replacement of the *Eag*I and *Bam*HI fragments was done as described above*.*

Plasmid construction of pET-*SopfaA-S1*

A DNA fragment carrying *hlyB* sequence S1 (accession number NC_004347, 118^th^ to 224^th^ amino acids of HlyB, Supplementary Table 4), which is an ABC transporter from *S. oneidensis*, was amplified by PCR with primers (SP52/SP53) and the *S. oneidensis* genome as a template. The fragment was digested with *Pvu*II and *Eag*I, and inserted into the *Afe*I and *Eag*I sites of pHSG298-ACP2. The replacement of the *Eag*I and *Bam*HI fragments was done as described above*.*

Plasmid construction of pET-*SofaA-S2*

A DNA fragment carrying *hlyB* sequence S2 (accession number NC_004347, 383^rd^ to 489^th^ amino acids of HlyB, Supplementary Table 4) was amplified by PCR with primers (SP54/SP55) and the *S. oneidensis* genome as a template. After this, the protocol for pET-*SopfaA-S1* was employed.

**References**

1. Hauvermale, A. *et al.* Fatty acid production in *Schizochytrium* sp.: involvement of a polyunsaturated fatty acid synthase and a type I fatty acid synthase. *Lipids* **41**, 739–747 (2006)
2. Amann, E., Ochs, B. & Abel, K. J. Tightly regulated *tac* promoter vectors useful for the expression of unfused and fused proteins in *Escherichia coli*. *Gene* **69**, 301–315 (1988)
3. Ujihara, T., Nagano, M., Wada, H. & Mitsuhashi, S. Identification of a novel type of polyunsaturated fatty acid synthase involved in arachidonic acid biosynthesis. *FEBS Lett.* **588**, 4032–4036 (2014)
